# Combination of Heme Oxygenase-1 Inhibition and Sigma Receptor Modulation for Anticancer Activity

**DOI:** 10.3390/molecules26133860

**Published:** 2021-06-24

**Authors:** Giuseppe Romeo, Valeria Ciaffaglione, Emanuele Amata, Maria Dichiara, Loredana Calabrese, Luca Vanella, Valeria Sorrenti, Salvo Grosso, Agata Grazia D’Amico, Velia D’Agata, Sebastiano Intagliata, Loredana Salerno

**Affiliations:** 1 Department of Drug and Health Sciences, University of Catania, viale A. Doria 6, 95125 Catania, Italy; gromeo@unict.it (G.R.); eamata@unict.it (E.A.); mariadic.md@gmail.com (M.D.); loredanacalabrese94@gmail.com (L.C.); lvanella@unict.it (L.V.); sorrenti@unict.it (V.S.); salvogrosso@outlook.it (S.G.); agata.damico@unict.it (A.G.D.); l.salerno@unict.it (L.S.); 2 Department of Biomedical and Biotechnological Sciences, University of Catania, 95100 Catania, Italy; vdagata@unict.it

**Keywords:** heme oxygenase, HO-1 inhibitors, sigma receptors, σR ligands, DU145, anticancer activity, combination therapy, hybrid compounds

## Abstract

Cancer is a multifactorial disease that may be tackled by targeting different signaling pathways. Heme oxygenase-1 (HO-1) and sigma receptors (σRs) are both overexpressed in different human cancers, including prostate and brain, contributing to the cancer spreading. In the present study, we investigated whether HO-1 inhibitors and σR ligands, as well a combination of the two, may influence DU145 human prostate and U87MG human glioblastoma cancer cells proliferation. In addition, we synthesized, characterized, and tested a small series of novel hybrid compounds (HO-1/σRs) **1**–**4** containing the chemical features needed for HO-1 inhibition and σR modulation. Herein, we report for the first time that targeting simultaneously HO-1 and σR proteins may be a good strategy to achieve increased antiproliferative activity against DU145 and U87MG cells, with respect to the mono administration of the parent compounds. The obtained outcomes provide an initial proof of concept useful to further optimize the structure of HO-1/σRs hybrids to develop novel potential anticancer agents.

## 1. Introduction

Despite a large number of molecules approved as anticancer drugs, cancer remains a serious cause of death worldwide [1]. Moreover, most antineoplastic agents currently in clinical practice have developed severe side effects along with multidrug resistance [2,3]. Therefore, the identification of new molecules, acting at classical or innovative molecular targets, which may improve or restore the effects of anticancer drugs, is of general interest.

Cancer can be considered, in all respects, a multi-genic disease. Cancer initiation and progression depend on more than one receptor or signaling pathway [4], suggesting that multi-targeted therapies may be advantageous over mono-therapy [5]. To date, diseases with complex etiologies such as cancer are often treated with “drug-cocktails” combining two or more molecules acting at different molecular targets, to optimize clinical response [6]. Combination therapy is generally associated with some drawbacks, such as the risk of metabolic interactions between drugs, and low patient compliance, due to multiple intakes during the day [7]. In order to overcome these negative aspects, an emerging strategy in medicinal chemistry is the search for novel bioactive compounds which combine in one molecule multi-target properties, called multi-target ligands or hybrid compounds [8,9].

Among the numerous pathways involved in the formation, growth, and survival of cancer cells, the heme oxygenase (HO) system and sigma receptors (σRs) play pivotal roles [10,11]. HO is the enzyme responsible for the oxidative catabolism of the pro-oxidant heme into free iron, carbon monoxide and biliverdin, subsequently reduced to bilirubin [12]. Two main isoforms catalyze this reaction in humans: HO-1 and HO-2. HO-2 is constitutive, ubiquitous, and mainly responsible for physiological effects [13]. HO-1 is inducible and expressed at basal levels only in the spleen and the liver, but it may be induced in many other organs and tissues by various stimuli, including heat, heme itself, heavy metals, ROS, and xenobiotics [14]. It has been widely acknowledged that HO-1 is a cytoprotective enzyme particularly active against oxidative stress [15,16]. However, HO-1 also exerts protective effects towards cancer cells, and aberrant high levels of HO-1 have been frequently reported in different human cancers, including prostate, lung, and pancreatic cancer, neuroblastoma, and chronic and acute myeloid leukemia. In addition, HO-1 overexpression has been associated with drug-resistance towards commonly used cancer therapies [17,18,19]. Thus, it is conceivable that HO-1 inhibitors can be useful in anticancer treatment either alone or in combination with chemotherapy, radiotherapy, or photodynamic therapy, according to recent literature reports [20,21,22].

σRs were earlier discovered as a subclass of opioid receptors [23]. Based on the current knowledge, σRs are considered non-opioid, non-GPCR transmembrane proteins expressed mainly in the endoplasmic reticulum (ER) membrane, classified into two subtypes: σ_1_R and σ_2_R/TMEM97. From their discovery, these receptors have attracted the attention of pharmacologists and medicinal chemists due to their pleiotropic functions on mitochondrial metabolism, apoptosis, ion channels modulation, lipid transport and metabolism regulation, neuritogenesis, mediation of Ca^2+^ release, and interplays with G protein-coupled receptors (GPCRs) [24]. As a consequence, these receptors are involved in several pathological conditions, including SNC disorders (neuropathic pain, depression, Alzheimer’s, Parkinson’s) [25,26], and expressed in many types of cancer cells (e.g., prostate, breast, colorectal cancer, glioblastoma) [27,28]. Indeed, both the σ_1_R and the σ_2_R might have a critical role in cancer growth, cell proliferation, and tumor aggressiveness [29]. In recent years, many selective or mixed σR ligands with potential biological effects have been developed [30,31,32,33]. It is generally accepted that, for anticancer activity, antagonism at σ_1_R or agonism at σ_2_R are preferable [34,35].

**Figure 1:**
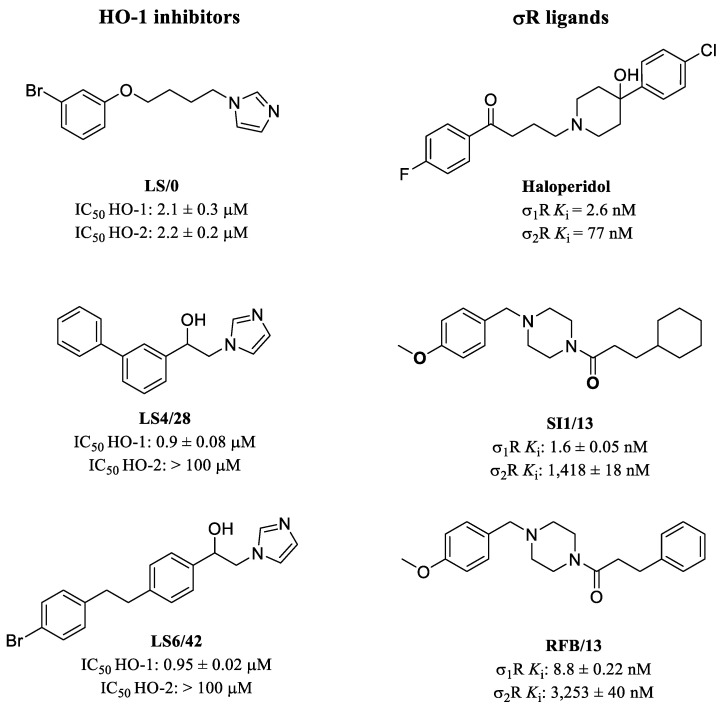
Structures of selected HO-1 inhibitors and σR ligands and their potency against respective targets.

**Figure 2:**
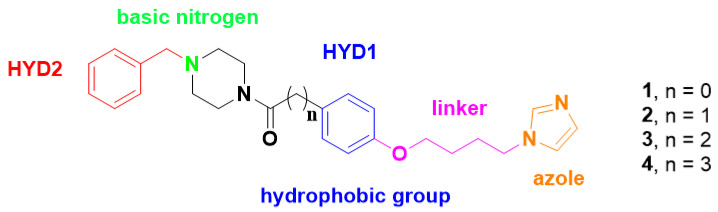
Structural requirements for both targets in HO-1/σRs hybrids **1**–**4** are depicted in different colors. For HO-1 inhibitors: azole nucleus (**orange**), hydrophobic group (**blue**), and central linker (magenta). For σR ligands: first hydrophobic portion HYD1 (**blue**), second hydrophobic portion HYD2 (**red**), and basic nitrogen (**green**).

On these premises, this work aimed to evaluate whether HO-1 inhibitors and σR ligands, as well as a combination of the two, may counteract cancer cell proliferation. In this regard, we selected DU145 and U87MG cells as representative cell lines for humane prostate cancer and glioblastoma, respectively, in which both HO-1 and σRs are involved [36,37,38,39]. Among HO-1 inhibitors, which we sourced from our library of compounds [40], we chose **LS/0**, **LS4/28,** and **LS6/42** as lead molecules, since it has been previously demonstrated that they are endowed with a very good HO inhibition profile (Figure 1), as well as antitumor properties [21,41,42,43,44]. Regarding σR ligands, we selected haloperidol (Figure 1) as an example of mixed σR ligand with anticancer activity against some tumors [45] and benzylpiperazine derivatives **SI1/13** and **RFB/13**, which emerged from our recent studies as very potent σ_1_R ligands, and very selective over the σ_2_R (Figure 1) [46]. Then, we synthesized new HO-1/σRs hybrid compounds **1**–**4**, which contain the structural requirements for interacting with both targets i.e., an azole-based moiety and a hydrophobic group connected by a central linker for HO-1, and a basic cyclic amine such as piperazine linked to two different hydrophobic moieties for σRs (Figure 2) [41,47]. The novel hybrids were tested to evaluate their capacity to inhibit HO-1, their affinity for σ_1_R and σ_2_R, and their cytotoxicity against DU145 and U87MG cancer cell lines.

## 2. Results and Discussions

### 2.1. Chemistry

**Scheme 1:**
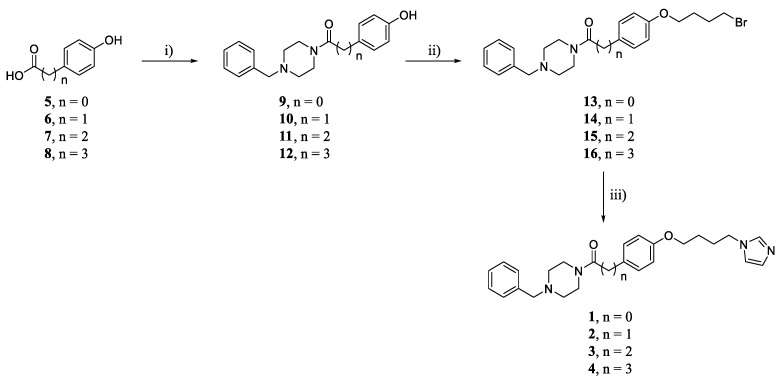
Reagents and conditions: (i) benzylpiperazine, CDI, THF, 0 ℃ 10 min, room temperature, 8 h; (ii) 1,4-dibromobutane, acetonitrile, K_2_CO_3_, reflux, 6 h; (iii) imidazole, THF, NaH, reflux, 9 h.

The synthesis of compounds **1**–**4** was accomplished in three steps, as shown in Scheme 1. The first step was the formation of the amides **9**–**12** obtained by means of a reaction between benzylpiperazine and a carboxylic acid derivative **5**–**8**, activated using 1,1′-carbonyldiimidazole (CDI). The following step was the etherification of **9**–**12** with 1,4–dibromobutane in acetonitrile under reflux and in presence of K_2_CO_3_, to give the corresponding bromobutoxy amides **13**–**16**. The final hybrids **1**–**4** were obtained through nucleophilic displacement of intermediates **13**–**16** with imidazole in THF, using sodium hydride (NaH) as base.

### 2.2. Biological Activity

#### 2.2.1. HO-1 Inhibition

The new HO-1/σRs hybrids **1**–**4** were tested to evaluate their HO-1 inhibitory activity. The enzyme was obtained from the microsomal fractions of rat spleen. Determination of HO-1 activity was performed by measuring the bilirubin formation using the difference in absorbance at 464–530 nm, as reported in the experimental section. Compounds **LS/0**, **LS4/28**, **LS6/42** and azalanstat were used as reference substances. The inhibitory potency is expressed as IC_50_ (µM) and results are shown in Table S1. We focused our attention on HO-1, since only this inducible isoform is involved in tumorigenesis and in tumor progression [48].

Structure–activity relationship (SAR) studies performed so far have evidenced that the pharmacophore for HO-1 inhibition contains three main critical portions: (i) an azole nucleus, preferably an imidazole, (ii) a hydrophobic moiety, and (iii) a central linker connecting the imidazole and the hydrophobic groups (Figure 2) [14]. Each chemical portion interacts with a specific amino acid located in three principal regions of the enzyme, near to the catalytic site which binds the substrate heme, and known as the eastern, western, and central regions, respectively, as described in crystallographic studies [49]. Many studies have demonstrated that the western region, responsible for binding with the hydrophobic portions of the inhibitors, is very flexible and can allocate both simple aromatic moieties, such as the phenyl group of reference compound **LS/0**, but also hindered, heteroaromatic, and ramified aryl moieties [50,51]. Then, using **LS/0** as a lead compound, we thought to increase molecular complexity by introducing a further moiety, i.e., benzyl piperazine, as this feature is necessary to target σRs. To find multiple contact points with the protein, we connected the benzylpiperazine with the phenoxybutylimidazole through a chain containing from 1 to 4 carbon atoms, then allowing free rotation, and, consequently, many possible different conformations. Unfortunately, all new hybrids are less potent than reference substances. It is likely that the western region of the enzyme, formed only by hydrophobic aminoacids, does not tolerate the presence of polar amino group, such as the *N*-atom belonging to the benzylpiperazine moiety present in hybrids, charged at physiological pH. Among hybrids **1**–**4**, compound **4**, containing the longest chain between benzylpiperazine and phenoxybutylimidazole, is the most potent compound among the series (Table S1), suggesting that a major distance of the piperazine ring allows a better binding of the first hydrophobic portion inside the western region of the enzyme.

#### 2.2.2. σRs Binding Properties

All new synthesized HO-1/σ hybrids **1**–**4** were evaluated for affinity at both the σ_1_R and the σ_2_R, through radioligand binding assay. Compounds **1**–**4** were designed taking into account pharmacophoric features for σRs affinity. Specifically, σR ligands require two hydrophobic moieties located at an appropriate distance from a basic nitrogen (HYD1, HYD2, and basic N, Figure 2), in order to properly allocate into the σR binding site [52]. In our σ_1_R lead compounds, **SI1/13** and **RFB/13**, the benzyl group linked to piperazine represents HYD2, the N-4 of piperazine is the basic nitrogen, and the other phenyl ring acts as HYD1. These moieties were maintained in our HO-1/σRs hybrids **1**–**4**; while an imidazole linked by an oxybutyl chain was further added with the aim of also targeting the HO-1 enzyme. Moreover, the choice of the benzylpiperazine moiety as σR pharmacophoric portion was further supported by the recent discovery of our highly potent and selective σ_1_R antagonist **SI1/13** [46]. Unexpectedly, none of the novel hybrids showed σRs affinity (Table S1), suggesting that the additional oxybutyl chain and the distal imidazole, with respect to **SI1/13** and **RFB/13**, are not tolerated by both σRs. According to Glennon’s pharmacophoric σ_1_R model [52], HYD1 can tolerate bulky groups; however, the presence of a flexible 4-(imidazolyl)butoxy group as a substituent might interfere through a steric hindrance in establishing essential hydrophobic interaction between the phenyl ring (located at the HYB1) and key amino acid residues inside the binding pockets.

#### 2.2.3. Cytotoxicity against DU145 and U87MG Cell Lines

In some types of human prostate and brain cancers, both HO-1 and σRs are overexpressed, influencing cancer cell proliferation. Consequently, σR ligands and HO-1 inhibitors may have anticancer activity. Literature data highlight the potential antiproliferative activity of various σ_1_R ligands in highly diffusive glioblastoma and prostate cancer cells, likely by interfering with the progression of cell cycle and decreasing the migration of cancer cells [11,28]. Likewise, HO-1 inhibition hinders cancer progression, mainly by decreasing CO-mediated angiogenesis and disrupting the antioxidant HO-1 activity [22,36,53]. In this study, we selected DU145 cells as representative for prostate cancer [37], and U87MG as cancer cells of glioblastoma [39], to evaluate whether inhibition of HO-1 and modulation of σRs may be effective for anticancer activity. Cytotoxicity of all new and previous synthesized compounds were evaluated in vitro via MTT assay. The cell viability of DU145 and U87-MG cells was assessed after 72 h of continuous treatment with all tested compounds, at the concentrations of 1, 10, and 50 μM.

**Figure 3:**
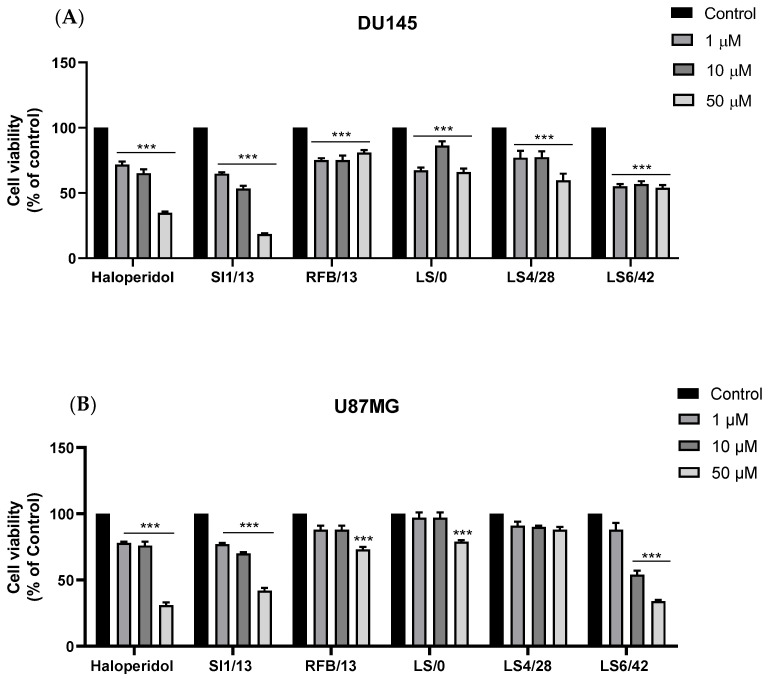
Effect of σR ligands haloperidol, **SI1/13** and **RFB/13** and of HO-1 inhibitors **LS/0**, **LS4/28** and **LS6/42** treatments on cell viability of DU145 (panel **A**) and U87MG (panel **B**) cell lines, assessed by MTT assay at the doses of 1, 10 and 50 μM. Results are representative of at least three independent experiments and values are expressed as percentage of control (% of control). Data represent means ± SEM. *** *p* < 0.001 vs. control as determined by One-way ANOVA followed by Tukey’s multiple comparison test.

Firstly, we tested the cytotoxicity against both cell lines of the σR_1_ ligands, **SI1/13** and **RFB/13**, and HO-1 inhibitors **LS/0**, **LS4/28**, and **LS6/42** (Figure 3, panels A and B). Among σR ligands we also included haloperidol, an antipsychotic drug endowed with antitumor properties due to its affinity for both σ_1_R and σ_2_R. The results in Figure 3A show that both σR ligands and HO-1 inhibitors were able to reduce DU145 cells proliferation with a different range of potency. In particular, haloperidol, the σR_1_ ligand **SI1/13**, and the HO-1 inhibitor **LS6/42** were the most potent, since at 10 μM they reduced the cell viability of about 50%. The same trend was observed in U87MG glioblastoma cells. In fact, as displayed in Figure 3B, **SI1/13** showed high efficacy in downregulation of cell viability, similarly to haloperidol, at all concentrations tested, whereas **RFB/13** and **LS/0** were able to significantly reduce cell viability only at 50 μM. **LS4/28** did not affect U87MG cell viability; on the contrary, **LS6/42** was the most efficacious since they reduced cellular viability by about 50% at 10 μM.

**Figure 4:**
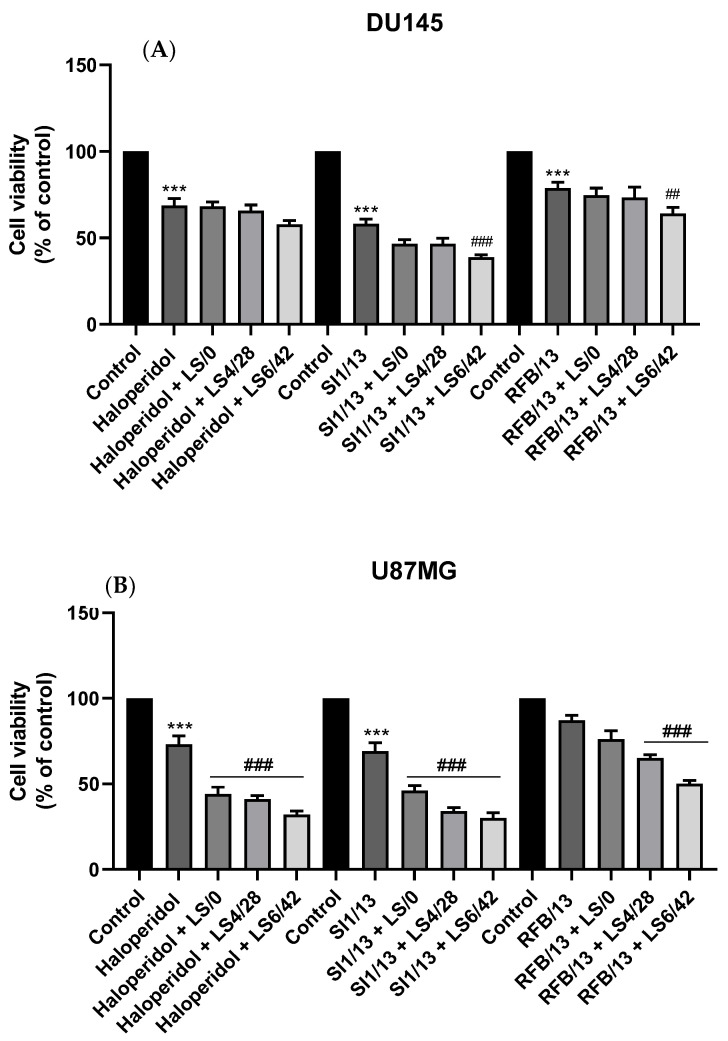
Effects of the combination of σR ligands haloperidol, **SI1/13** and **RFB/13** and of HO-1 inhibitors **LS/0**, **LS4/28** and **LS6/42** treatments on cell viability of DU145 (panel **A**) and U87MG (panel **B**) cell lines, assessed by MTT at the doses of 10 μM, and compared to the effect obtained with σR ligands alone at the same dose. Results are representative of at least three independent experiments and values are expressed as percentage of control (% of control). Data represent means ± SEM. *** *p* < 0.001 vs. control, ^##^ *p* < 0.001 and ^###^ *p* < 0.001 vs. σR ligand as determined by one-way ANOVA followed by Tukey’s multiple comparison test.

Since HO-1 and σRs are both involved in examined cancers, we wanted to evaluate whether a simultaneous treatment with σR ligands and HO-1 inhibitors may have some advantage with respect to single compounds. In this regard, we combined 10 μM of haloperidol, **SI1/13**, or **RFB/13** with the same amount of **LS/0**, **LS4/28**, or **LS6/42**. Results are shown in Figure 4A for DU145 cells and in Figure 4B for U87MG cells, respectively. Combination of the σR ligand **SI1/13** with HO-1 inhibitors, in particular **LS6/42**, was noteworthy in DU145 cells; in fact, 10 μM of **SI1/13** plus 10 μM of **LS6/42** reduced cell viability of about 75% with respect to the 50% effects showed by the single compounds. The effect of σR ligands and HO-1 inhibitors co-administration was noteworthy in U87MG cells, where all the combinations afforded to reduced cell proliferation with respect to that obtained with single compounds. Specifically, the viability was significantly reduced for compound **RFB/13** only when combined with **LS4/28** or **LS6/42**, whereas the antiproliferative action of haloperidol and **SI1/13** was increased by the addition of all the tested HO-1 inhibitors. The most efficacious combinations were haloperidol plus **LS6**/**42** and **SI1/13** plus **LS6/42**.

**Figure 5:**
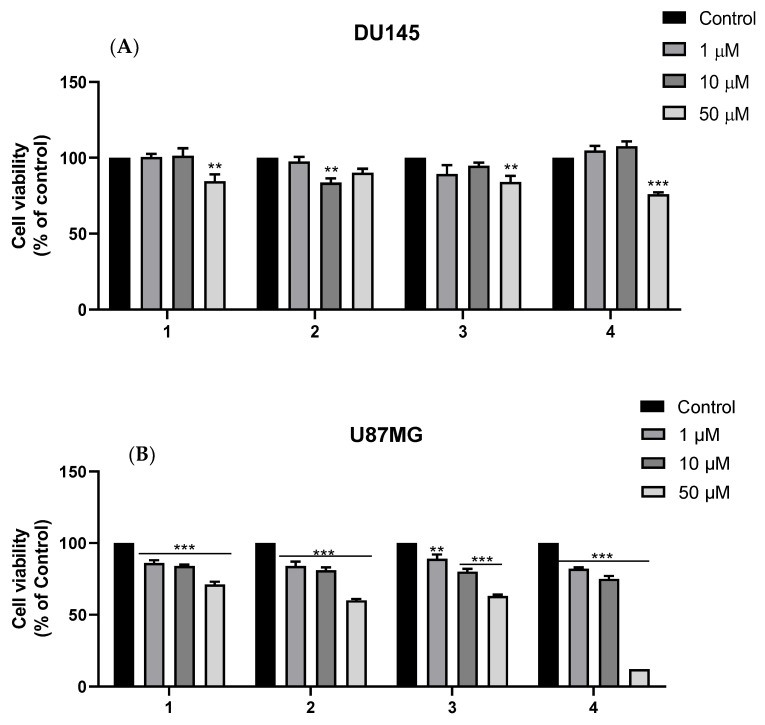
Effect of HO-1/σR hybrids **1**–**4** treatments on cell viability of DU145 (panel **A**) and U87MG (panel **B**) cell lines, assessed by MTT assay at the doses of 1, 10 and 50 μM. Results are representative of at least three independent experiments and values are expressed as percentage of control (% of control). Data represent means ± SEM. ** *p* < 0.01, *** *p* < 0.001 vs. control as determined by one-way ANOVA followed by Tukey’s multiple comparison test.

Finally, we tested the viability of DU145 and U87MG cancer cells in the presence of all new HO-1/σRs hybrids **1**–**4**. Results showed in Figure 5A evidence that the new compounds **1**–**4** were able to influence cell proliferation of DU145 cell line only at high concentrations. Glioblastoma U87MG cancer cells became more sensitive after the treatment with hybrids **1**–**4**. In fact, as showed in Figure 5B, compounds **1**, **2** and **4** reduced U87MG cell viability at all concentrations, especially at 50 μM, compared to control group; instead, compound **3** showed less efficacy than the control at 1 μM.

The low cytotoxicity against DU145 cells and the moderate antiproliferative activity towards U87MG cells of HO-1/σRs hybrids **1**–**4** correlate well to the low potency towards both HO-1 and σRs proteins showed by the same compounds **1**–**4**. Nevertheless, an encouraging reduction in the viability of both cancer cells was obtained after co-administration of HO-1 inhibitors and σR ligands parent molecules, confirming that simultaneous inhibition of HO-1 and modulation of σRs may be a valuable target for anticancer activity.

## 3. Materials and Methods

### 3.1. Chemistry

Melting points were determined by using an Electrothermal IA9200 apparatus containing a digital thermometer. Determinations were achieved after introducing glass capillary tubes, filled with analytes, inside the apparatus, and are uncorrected. ^1^H NMR and ^13^C NMR spectra were recorded on Varian Inova Unity (200 MHz) spectrometers in DMSO-*d6* or CDCl_3_ solution. Chemical shifts are given in δ values to two digits after the decimal point in part per million (ppm), using tetramethylsilane (TMS) as the internal standard; coupling constants (*J*) are given in Hz. Signal multiplicities are indicated with the following abbreviations: s (singlet), d (doublet), t (triplet), q (quartet), m (multiplet), and br (broad signal). The IR spectra were recorded in KBr disks or Neat, on a Perkin Elmer 1600 series FT-IR spectrometer. Reactions were monitored by thin-layer chromatography (TLC), carried out on Merck plates (Kieselgel 60 F254), using UV light (254 and 366 nm) for visualization and developed using iodine chamber. Flash column chromatography was performed on Merck silica gel 60 0.040–0.063 mm (230–400 mesh). Reagents, solvents and starting materials were purchased from commercial suppliers.

The synthetic procedures and characterization of intermediates **9**–**16** are described in the Supplementary Materials.

General procedure for the synthesis of (1*H*-imidazol-1-yl)butoxy phenyl ketones (**1**–**4**). NaH (2.54 mmol) was added to a solution of 1*H*-imidazole (1.52 mmol) in anhydrous THF (12 mL) under nitrogen. After 15 min, the appropriate bromobutoxy phenyl derivative (**13**–**16**), previously solubilized in THF (12 mL), was added and the reaction mixture was left stirring for 9 h under reflux. The solvent was evaporated under vacuum, then water (100 mL) was added to the resulting residue and extracted with ethyl acetate (3 × 50 mL). The organic layer was washed with a basic solution (NaOH 0.5N 20 mL), brine (50 mL), dried over anhydrous Na_2_SO_4_, filtered, and concentrated. The obtained residue was purified by flash column chromatography using ethyl acetate/methanol (9.5/ 0.5).

(4-(4-(1*H*-imidazol-1-yl)butoxy)phenyl)(4-benzylpiperazin-1-yl)methanone (**1**). Colorless oil: yield 96,39 %. IR (KBr, selected lines) cm^−1^: 3402, 2940, 1657, 1610, 1512, 1461, 1300, 1176, 1026, 842. ^1^H NMR (200 MHz, DMSO-*d*_6_): δ 7.64 (s, 1H, imidazole), 7.38–7.21 (m, 5H + 2H, aromatic), 7.19 (s, 1H, imidazole), 6.99–6.90 (m, 2H, aromatic), 6.89 (s, 1H, imidazole), 4.07–3.95 (m, 2H + 2H, O-C*H*_2_-CH_2_-CH_2_-C*H*_2_-N), 3.55–3.39 (m, 2H + 4H, Ar-C*H_2_*-N + piperazine), 2.42–2.31 (m, 4H, piperazine), 1.93–1.71 (m, 2H, O-CH_2_-C*H*_2_-CH_2_-CH_2_-N), 1.71–1.58 (m, 2H, O-CH*_2_*-CH_2_-C*H*_2_-CH_2_-N). ^13^C NMR (50 MHz, CDCl_3_): δ 170.2, 159.9, 137.4, 129.4, 129.2, 128.8, 128.7, 128.4, 128.1, 127.4, 118.8, 114.1, 67.2, 62.9, 53.1, 50.4, 46.8, 28.0, 26.2. Anal. Calcd. for (C_25_H_30_N_4_O_2_): C, 71.74; H, 7.23; N, 13.39. Found: C, 71.56; H, 7.21; N, 13.42.

2-(4-(4-(1*H*-imidazol-1-yl)butoxy)phenyl)-1-(4-benzylpiperazin-1-yl)ethan-1-one (**2**). Colorless oil: yield 72,95 %. IR (KBr, selected lines) cm^−1^: 2939, 2810, 1640, 1512, 1452, 1244, 1178, 1000, 742. ^1^H NMR (200 MHz, CDCl_3_): δ 7.53 (s, 1H, imidazole), 7.29 (s, 5H, aromatic), 7.18–7.02 (m, 3H, aromatic), 6.94 (s, 1H, imidazole), 6.81 (d, *J* = 8.5 Hz, 2H, aromatic + imidazole), 4.06–3.91 (m, 4H, O-C*H*_2_-CH_2_-CH_2_-CH_2_-N + O-CH_2_-CH_2_-CH_2_-C*H_2_*-N), 3.64–3.62 (m, 4H, CO-C*H*_2_-Ar + Ar-C*H_2_*-N), 3.47–3.41 (m, 4H, piperazine), 2.40 (t, *J* = 10 Hz, 2H, piperazine), 2.27 (t, *J* = 8 Hz, 2H, piperazine), 2.02–1.91 (m, 2H, O-CH*_2_*-CH_2_-C*H*_2_-CH_2_-N), 1.82–1.72 (m, 2H, O-CH*_2_*-C*H*_2_-CH_2_-CH_2_-N). ^13^C NMR (50 MHz, CDCl_3_): δ 169.8, 157.5, 137.5, 129.7, 129.3, 129.1, 128.7, 128.3, 127.3, 127.3, 118.8, 114.6, 67.1, 62.8, 52.9, 52.7, 46.8, 46.0, 41.8, 40.0, 28.1, 26.3. Anal. Calcd. for (C_26_H_32_N_4_O_2_): C, 72.19; H, 7.46; N, 12.95. Found: C, 71.98; H, 7.44; N, 12.99.

3-(4-(4-(1*H*-imidazol-1-yl)butoxy)phenyl)-1-(4-benzylpiperazin-1-yl)propan-1-one (**3**). Colorless oil: yield 60,49 %. IR (KBr, selected lines) cm^−1^: 3456, 2942, 1631, 1513, 1443, 1242, 825. ^1^H NMR (200 MHz, DMSO-*d*_6_): δ 7.66 (s, 1H, imidazole), 7.33–7.23 (m, 5H, aromatic), 7.19 (s, 1H, imidazole), 7.12 (d, *J* = 8.6 Hz, 2H, aromatic), 6.89 (s, 1H, imidazole), 6.81 (d, *J* = 8.6 Hz, 2H, aromatic), 4.02 (t, *J* = 7.0 Hz, 2H, O-C*H*_2_-CH_2_-CH_2_-CH_2_-N), 3.95 (t, *J* = 6.2 Hz, 2H, O-CH_2_-CH_2_-CH_2_-C*H_2_*-N), 3.45–3.35 (m, 2H + 4H, Ar-C*H_2_*-N + piperazine), 2.72 (t, *J* = 6.8 Hz, 2H, CO-CH_2_-C*H*_2_-Ar), 2.59–2.51 (m, 2H, CO-C*H*_2_-CH_2_-Ar), 2.27–2.51 (m, 4H, piperazine), 1.89–1.76 (m, 2H, O-CH*_2_*-C*H*_2_-CH_2_-CH_2_-N), 1.69–1.56 (m, 2H, O-CH*_2_*-CH_2_-C*H*_2_-CH_2_-N). ^13^C NMR (50 MHz, CDCl_3_): δ 170.7, 157.2, 137.5, 133.6, 129.5, 129.2, 128.7, 128.4, 127.4, 118.9, 118.9, 114.5, 67.2, 62.9, 53.0, 52.8, 46.9, 45.6, 41.6, 35.4, 30.7, 28.2, 26.4. Anal. Calcd. for (C_27_H_34_N_4_O_2_): C, 72.62; H, 7.67; N, 12.55. Found: C, 72.53; H, 7.66; N, 12.58.

4-(4-(4-(1*H*-imidazol-1-yl)butoxy)phenyl)-1-(4-benzylpiperazin-1-yl)butan-1-one (**4**). Orange oil: yield 85 %. IR (KBr, selected lines) cm^−1^: 3430, 2926, 1631, 1512, 1443, 1241, 1028, 999, 832, 744. ^1^H NMR (200 MHz, CDCl_3_) δ 7.67 (s, 1H, imidazole), 7.39–7.28 (m, 4H + 1H, aromatic + imidazole), 7.10 (d, *J* = 8.2 Hz, 3H, aromatic), 6.98 (s, 1H, imidazole), 6.81 (d, *J* = 8.4 Hz, 2H, aromatic), 4.08 (t, *J* = 7.2 Hz, 2H, O-C*H*_2_-CH_2_-CH_2_-CH_2_-N), 3.97 (t, *J* = 5.8 Hz, 2H, O-CH_2_-CH_2_-CH_2_-C*H_2_*-N), 3.74–3.62 (m, 2H, piperazine), 3.57 (s, 2H, Ar-C*H_2_*-N), 3.49–3.37 (m, 2H, piperazine), 2.62 (t, *J* = 7.5 Hz, 2H, piperazine), 2.52–2.37 (m, 4H, CO-C*H*_2_-CH_2_-C*H*_2_-Ar), 2.31 (t, *J* = 7.5 Hz, 2H, piperazine), 2.00–1.86 (m, 2H + 2H, O-CH*_2_*-CH_2_-C*H*_2_-CH_2_-N + CO-CH_2_-C*H*_2_-CH_2_-Ar), 1.88–1.69 (m, 2H, O-CH*_2_*-C*H*_2_-CH_2_-CH_2_-N). ^13^C NMR (50 MHz, CDCl_3_): δ 171.3, 157.0, 137.3, 134.0, 129.5, 129.4, 129.2, 128.7, 128.4, 127.4, 114.3, 67.1, 62.9, 53.1, 52.8, 47.0, 45.5, 41.5, 34.5, 32.4, 28.1, 27.0, 26.3. Anal. Calcd. for (C_28_H_36_N_4_O_2_): C, 73.01; H, 7.88; N, 12.16. Found: C, 72.91; H, 7.87; N, 12.20.

### 3.2. Biology

#### 3.2.1. Preparation of Spleen Microsomal Fractions

Microsomal fractions obtained from rat spleen were used as sources of HO-1. Microsomal preparations obtained by differential centrifugation, were selected in order to use the most native (i.e., closest to in vivo) forms of HO-1. The experiments reported in the present paper complied with current Italian law and met the guidelines of the Institutional Animal Care and Use Committee of Ministry of Health (Directorate General for Animal Health and Veterinary Medicines) (Italy). The experiments were performed in male Sprague–Dawley albino rats (150 g body weight and age 45 d). They had free access to water and were kept at room temperature with a natural photo-period (12 h light, 12 h dark cycle). For measuring HO-1 activities, each rat was sacrificed and their spleens were excised and weighed. A homogenate (15%, *w*/*v*) of spleens pooled from four rats was prepared in ice-cold HO-homogenizing buffer (50 mM Tris buffer, pH 7.4, containing 0.25 M sucrose) using a Potter–Elvehjem homogenizing system with a Teflon pestle. The microsomal fraction of rat spleen homogenate was obtained by centrifugation at 10,000× *g* for 20 min at 4 °C, followed by centrifugation of the supernatant at 100,000× *g* for 60 min at 4 °C. The 100,000g pellet (microsomes) was resuspended in an 100 mM potassium phosphate buffer, pH 7.8, containing 2 mM MgCl_2_ with a Potter–Elvehjem homogenizing system. The rat spleen microsomal fractions were divided into equal aliquots, placed into microcentrifuge tubes, and stored at −80 °C for up to 2 months.

#### 3.2.2. Preparation of Biliverdin Reductase

Biliverdin reductase was obtained from liver cytosol. Rat liver was perfused through the hepatic portal vein with cold 0.9% NaCl, then it was cut and flushed with 2 × 20 mL of ice-cold PBS to remove all of the blood. Liver tissue was homogenized in 3 volumes of solution containing 1.15% KCl *w*/*v* and Tris buffer 20 mM, pH 7.8 on ice. Homogenates were centrifuged at 10,000× *g*, for 20 min at 4 °C. Supernatant was decanted and centrifuged at 100,000× *g* for 1 h at 4 °C to sediment the microsomes. The 100,000× *g* supernatant was saved and then stored in small amounts at −80 °C after its protein concentration was measured.

#### 3.2.3. Measurement of HO-1 Enzymatic Activity in Microsomal Fraction of Rat Spleen

The HO-1 activities were determined by measuring the bilirubin formation using the difference in absorbance at 464–530 nm. Reaction mixtures (500 µL) contained 20 mM Tris–HCl, pH 7.4, (1 mg/mL) microsomal extract, 0.5–2 mg/mL biliverdin reductase, 1 mM NADPH, 2 mM glucose 6-phosphate (G6P), 1 U G6P dehydrogenase, 25 μM hemin, and 10 µL of DMSO (or the same volume of DMSO solution of test compounds to a final concentration of 100, 10, and 1 µM). Samples were incubated for 60 min at 37 °C in a circulating water bath in the dark. Reactions were stopped by adding the same volume of chloroform. After recovering the chloroform phase, the amount of bilirubin formed was measured with a double-beam spectrophotometer as OD464–530 nm (extinction coefficient, 40 mM/cm^−1^ for bilirubin). One unit of the enzyme was defined as the amount of enzyme catalyzing the formation of 1 nmol of bilirubin/mg protein/h.

#### 3.2.4. Radioligand Binding Assay

Brain and liver homogenates for σ_1_R and σ_2_R binding assays were prepared from male Dunkin–Hartley guinea pigs and Sprague–Dawley rats, respectively, (ENVIGO RMS S.R.L., Udine, Italy) as previously reported [54]. In vitro σ_1_R ligand binding assays were carried out in Tris buffer (50 mM, pH 7.4) for 150 min at 37 °C. The thawed membrane preparation of guinea pig brain cortex was incubated with increasing concentrations of test compounds and [^3^H](+)-pentazocine (2 nM) in a final volume of 0.5 mL. Unlabeled (+)-pentazocine (10 μM) was used to measure non-specific binding. Bound and free radioligand were separated by fast filtration under reduced pressure using a Millipore filter apparatus through Whatman GF 6 glass fiber filters, which were presoaked in a 0.5% poly(ethyleneimine) water solution. Each filter paper was rinsed three times with ice-cold Tris buffer (50 mM, pH 7.4), dried at rt, and incubated overnight with scintillation fluid into pony vials. The bound radioactivity has been determined using a liquid scintillation counter (Beckman LS 6500) [55]. In vitro σ_2_R ligand binding assays were carried out in Tris buffer (50 mM, pH 8.0) for 120 min at rt. The thawed membrane preparation of rat liver was incubated with increasing concentrations of test compounds and [^3^H]DTG (2 nM) in the presence of (+)-pentazocine (5 µM) as σ_1_R masking agent in a final volume of 0.5 mL. Non-specific binding was evaluated with unlabeled DTG (10 μM). Bound and free radioligand were separated by fast filtration under reduced pressure using a Millipore filter apparatus through Whatman GF 6 glass fiber filters, which were presoaked in a 0.5% poly(ethyleneimine) water solution. Each filter paper was rinsed three times with ice-cold Tris buffer (10 mM, pH 8), dried at rt, and incubated overnight with scintillation fluid into pony vials. The bound radioactivity was determined using a liquid scintillation counter (Beckman LS 6500) [56]. The *K*_i_-values were calculated with the program GraphPad Prism^®^ 7.0 (GraphPad Software, San Diego, CA, USA). The *K*_i_-values are given as mean value ± SD from at least two independent experiments performed in duplicate.

#### 3.2.5. Cell Cultures

Two lines of cancer cells were used to conduct our investigations. In particular, we used the human glioblastoma cell line U87MG (ATCCC number #HTB-14) and the prostate cancer cell line DU145 (ATCC HTB-81). These cell lines were obtained from the American Type Culture Collection (ATCC, Rockville, Md., USA). Cells were cultured in Dulbecco’s modified Eagle’s medium (DMEM) supplemented with 10% of heat-inactivated fetal bovine serum (FBS), 100 U/mL penicillin and 100-μg/mL streptomycin (Sigma-Aldrich, Steinheim, Germany) and incubated at 37 °C in a humidified atmosphere with 5% CO_2_.

#### 3.2.6. In Vitro Cytotoxicity of HO-1 Inhibitors, σR Ligands, and HO-1/σR Hybrids 1–4 against DU145 and U87MG Cancer Cell Lines

The cytotoxicity of HO-1 inhibitors and σR ligands previous synthesized compounds as well as novel HO-1/σR hybrids compounds were evaluated. The effect on cell viability was assessed by performing MTT assay. Cells were seeded into 96-well plates at a density of 7.0 × 10^3^ cells/well in 100 µL of culture medium. The day after, cells were treated with each molecule at three different concentrations (1 µM, 10 µM and 50 µM) for 72 h. Following treatments, 0.5 mg/mL of 3-[4,5-dimethylthiazol-2-yl]-2,5-diphenyltetrazolium bromide (MTT) (Sigma-Aldrich) was added to each well and incubated for 4 h at 37 °C. Finally, dimethyl-sulfoxide (DMSO) was used to dissolve formazan salts and absorbance was measured at 450 nm in a microplate reader (Biotek Synergy-HT). Six replicate wells were used for each group.

## 4. Conclusions

Due to its complex etiology, cancer may be counteracted by targeting different biological pathways. In this paper, we investigated whether the inhibition of HO-1 enzymatic activity and simultaneous modulation of the σR functions may have some advantages in reducing the proliferation of DU145 human prostate and U87MG glioblastoma cancer cell lines. In this regard, compounds alone, a combination of two compounds, i.e., σR ligands haloperidol, **SI1/13** or **RFB/13** plus HO-1 inhibitors **LS/0**, **LS4/28** or **LS6/42**, and HO-1/σRs hybrid compounds **1**–**4** were evaluated. Although hybrids **1**–**4** showed only moderate antiproliferative activity against glioblastoma cells, we proved for the first time that simultaneously targeting HO-1 and σR proteins reduces DU145 and U87MG cell proliferation to a major extent concerning the effect achieved with single compounds. The obtained results serve as an initial proof of concept, useful for optimizing HO-1/σRs hybrids’ structure to develop novel potential anticancer agents.

## Supplementary Materials

The following are available online at: Synthesis of intermediates **9**–**12**. Synthesis of intermediates **13**–**16**. Table S1: HO-1 inhibition and binding properties of hybrids **1**–**4** and reference compounds. Table S2: Elemental analysis data for compounds **1**–**4.** Figures S1–S8: ^1^H NMR spectra of intermediates **9**–**16**. Figures S9-S16: ^1^H NMR and ^13^C NMR spectra of compounds **1**–**4**. Figure S17: Viability of MDA-MB 231 cell line, References.
