# Supplementary material for: Combination of Heme Oxygenase-1 Inhibition and Sigma Receptor Modulation for Anticancer Activity
Source: Molecules. 2021 Jun 24;26(13):3860. doi: 10.3390/molecules26133860 (PMC8270315; doi:10.3390/molecules26133860)
Supplement: Supplementary file 1 [file molecules-26-03860-s001.zip › molecules-1240979-supplementary.pdf]

Supplementary Material

Combination of Heme Oxygenase-1 Inhibition and Sigma Receptor Modulation for Anticancer Activity

Giuseppe Romeo [1], Valeria Ciaffaglione [1,*], Emanuele Amata [1], Maria Dichiara [1], Loredana Calabrese [1], Luca Vanella [1], Valeria Sorrenti [1], Salvo Grosso [1], Agata Grazia D'Amico [1], Velia D'Agata [2], Sebastiano Intagliata [1,*] and Loredana Salerno [1]

[1] Department of Drug and Health Sciences, University of Catania, viale A. Doria 6, 95125 Catania, Italy; gromeo@unict.it (G.R.); eamata@unict.it (E.A.); mariadic.md@gmail.com (M.D.); loredanacalabrese94@gmail.com (L.C.); lvanella@unict.it (L.V.); sorrenti@unict.it (V.S.); salvogrosso@outlook.it (S.G.); agata.damico@unict.it (A.G.D.); l.salerno@unict.it (L.S.)

[2] Department of Biomedical and Biotechnological Sciences, University of Catania, 95100 Catania, Italy; vdagata@unict.it

* Correspondence: valeria.ciaffaglione@phd.unict.it (V.C.); s.intagliata@unict.it (S.I.); Tel.: +39-095-738-4006 (V.C.); +39-095-738-4053 (S.I.).

Table of contents

**Synthesis of intermediates 9–12**	S2
**Synthesis of intermediates 13–16**	S3
**Table S1**: HO-1 inhibition and binding properties of hybrids **1–4** and reference compounds.	S4
**Table S2**: Elemental analysis data for compounds **1–4**.	S4
**Figures S1–S8**: ^{1}H NMR spectra of intermediates **9–16**.	S5-S8
**Figures S9–S16**: ^{1}H NMR and ^{13}C NMR spectra of compounds **1–4**.	S9-S12
**Figure S17**: viability of MDA-MB 231 cell line	S13
**References**	S13

General procedure for the synthesis of 1-(4-benzylpiperazin-1-yl)hydroxyphenyl derivatives (**9**–**12**)

4-hydroxybenzoic acid, 2-(4-hydroxyphenyl)acetic acid, 3-(4-hydroxyphenyl)propanoic acid, or 4-(4-hydroxyphenyl)butanoic acid (**5**–**8**, respectively) (6.02 mmol) was dissolved in THF anhydrous (10 mL). 1,1'-carbonyldiimidazole (CDI) (6.02 mmol) was added and the reaction mixture was left stirring under nitrogen for 10 min. Benzylpiperazine (6.02 mmol) was dissolved in THF anhydrous (5 mL) in a dropping funnel. The reaction mixture was cooled at 0 °C for 10 m. The benzylpiperazine was added dropwise and the reaction occurred at room temperature for 8 h. The solvent was evaporated under vacuum, and for compounds **9**–**11** the resulting yellow oil was washed with $NaHCO_3$ 0,5 % m/v aqueous solution (50 mL), and water (2 x 50 mL). Then, ethanol (10 mL) was added and volatiles were again eliminated under vacuum. For compound **12**, ethyl acetate (50 mL) was added to the yellow residue. The organic layer was then washed with saturated solution of $NaHCO_3$ (3 x 25 mL), dried over anhydrous Na_2SO_4, filtered, and concentrated. The obtained oily residue was purified by flash column chromatography using a mixture of dichloromethane/methanol or ethyl acetate/methanol (9.5/ 0.5).

(4-Benzylpiperazin-1-yl)(4-hydroxyphenyl)methanone (**9**)

White solid; mp: 170.2–173.5 °C; yield 34.64 %. IR (KBr, selected lines) cm^{-1}: 3196, 2948, 2810, 1734, 1608, 1578, 1434, 1364, 1277, 1169, 1003, 848. ^1H NMR (200 MHz, DMSO-d_6): δ 9.89 (s, 1H, OH phenolic), 7.39–7.19 (m, 5H + 2H, aromatic), 6.82–6.74 (m, 2H, aromatic), 3.58–3.25 (m, 2H + 4H, Ar-CH_2-N + piperazine), 2.43–2.32 (m, 4H, piperazine).

1-(4-Benzylpiperazin-1-yl)-2-(4-hydroxyphenyl)ethan-1-one (**10**)

White solid; mp: 156.3–157.4 °C; yield 26.84 %. IR (KBr, selected lines) cm^{-1}: 3319, 2914, 2814, 1631, 1516, 1437, 1410, 1354, 1264, 1208, 1003, 820. ^1H NMR (200 MHz, DMSO-d_6): δ 9.28 (s, 1H, OH phenolic), 7.36–7.24 (m, 5H, aromatic), 6.99 (d, *J* = 8.4 Hz, 2H, aromatic), 6.67 (d, *J* = 8.6 Hz, 2H, aromatic), 3.55 (s, 2H, CO-CH_2-Ar), 3.48–3.32 (m, 2H + 4H, Ar-CH_2-N + piperazine), 2.29–2.20 (m, 4H, piperazine).

1-(4-Benzylpiperazin-1-yl)-3-(4-hydroxyphenyl)propan-1-one (**11**)

White solid; mp: 107.8–109.2 °C; yield 28.6 %. IR (KBr, selected lines) cm^{-1}: 3376, 3023, 2805, 1643, 1514, 1454, 1346, 1212, 1026, 999, 812. ^1H NMR (200 MHz, DMSO-d_6): δ 9.19 (s, 1H, OH phenolic), 7.38–7.21 (m, 5H, aromatic), 7.00 (d, *J* = 8.2 Hz, 2H, aromatic), 6.64 (d, *J* = 8.2 Hz, 2H, aromatic), 3.42–3.30 (m, 4H, piperazine), 2.67–2.63 (m, 2H, CO-CH_2-CH_2-Ar), 2.54–2.50 (m, 2H, CO-CH_2-CH_2-Ar), 2.39–2.11 (m, 4H, piperazine).

1-(4-Benzylpiperazin-1-yl)-4-(4-hydroxyphenyl)butan-1-one (**12**)

White solid; mp: 145.3–146.9 °C; yield 47 %. IR (KBr, selected lines) cm^{-1}: 3285, 2951, 2910, 2822, 1623, 1595, 1452, 1348, 1257, 1147, 996, 835. ^1H NMR (200 MHz, $CDCl_3$): δ 7.38–7.35 (m, 5H, aromatic), 6.98 (d, *J* = 8.4 Hz, 2H, aromatic), 6.76 (d, *J* = 8.4 Hz, 2H, aromatic), 3.72–3.69 (m, 2H, piperazine), 3.64 (s, 2H, Ar-CH_2-N), 3.55–3.42 (m, 2H, piperazine), 2.64–2.43 (m, 4H + 2H, piperazine + CO-CH_2-CH_2-CH_2-Ar), 2.29 (t, *J* = 7.5 Hz, 2H, CO-CH_2-CH_2-CH_2-Ar), 2.02–1.80 (m, 2H, CO-CH_2-CH_2-CH_2-Ar).

General procedure for the synthesis of bromobutoxy phenyl derivatives (**13**–**16**)

The appropriate (4-benzylpiperazin-1-yl)hydroxyphenyl intermediate (**9**–**12**) (1.34 mmol) was dissolved in acetonitrile (25 mL). K_2CO_3 (5.36 mmol) was added and the reaction mixture was left stirring under reflux for 10 min. Then 1,4-dibromobutane (5.36 mmol) was added and the reaction completed in 6 h. The solvent was evaporated under vacuum, then water (100 mL) was added to the resulting residue and extracted with ethyl acetate (3 x 50 mL). The organic layer was finally washed with brine (50 mL) and dried over anhydrous Na_2SO_4, filtered, and concentrated. The obtained residue was purified by flash column chromatography using ethyl acetate/methanol (9.5/ 0.5).

(4-Benzylpiperazin-1-yl)(4-(4-bromobutoxy)phenyl)methanone (**13**)

Colorless oil; yield 81.20 %. ^{1}H NMR (200 MHz, DMSO-d_6): δ 7.36–7.31 (m, 5H + 2H, aromatic), 6.99–6.94 (m, 2H, aromatic), 4.03 (t, *J* = 5.2 Hz, 2H, O-C*H*$_2$-CH$_2$-CH$_2$-CH$_2$-Br), 3.61 (t, *J* = 6.4 Hz, 2H, O-CH$_2$-CH$_2$-CH$_2$-C*H*$_2$-Br), 3.47–3.41 (m, 2H + 4H, Ar-C*H*$_2$-N + piperazine), 2.39–2.35 (m, 4H, piperazine), 2.0–1.79 (m, 4H, O-CH$_2$-C*H*$_2$-C*H*$_2$-CH$_2$-Br).

1-(4-Benzylpiperazin-1-yl)-2-(4-(4-bromobutoxy)phenyl)ethan-1-one (**14**)

Yellow oil; yield 69.84 %. ^{1}H NMR (200 MHz, DMSO-d_6): δ 7.37–7.19 (m, 5H, aromatic), 7.10 (d, *J* = 8.4 Hz, 2H, aromatic), 6.85 (d, *J* = 8.6 Hz, 2H, aromatic), 3.98 (t, *J* = 6.2 Hz, 2H, O-C*H*$_2$-CH$_2$-CH$_2$-CH$_2$-Br), 3.72–3.56 (m, 2H + 2H, O-CH$_2$-CH$_2$-CH$_2$-C*H*$_2$-Br + CO-C*H*$_2$-Ar), 3.45 (s, 2H, Ar-C*H*$_2$-N), 3.40–3.25 (m, 4H, piperazine), 2.27–2.25 (m, 4H, piperazine), 1.99–1.78 (m, 4H, O-CH$_2$-C*H*$_2$-C*H*$_2$-CH$_2$-Br).

1-(4-Benzylpiperazin-1-yl)-3-(4-(4-bromobutoxy)phenyl)propan-1-one (**15**)

Colorless oil; yield 86.80 %. ^{1}H NMR (200 MHz, DMSO-d_6): δ 7.39–7.20 (m, 5H, aromatic), 7.12 (d, *J* = 8.6 Hz, 2H, aromatic), 6.82 (d, *J* = 8.6 Hz, 2H, aromatic), 3.98 (t, *J* = 6.0 Hz, 2H, O-C*H*$_2$-CH$_2$-CH$_2$-CH$_2$-Br), 3.60 (t, *J* = 6.4 Hz, 2H, O-CH$_2$-CH$_2$-CH$_2$-C*H*$_2$-Br), 3.45–3.36 (m, 2H + 4H, Ar-C*H*$_2$-N + piperazine), 2.80–2.67 (m, 2H, CO-CH$_2$-C*H*$_2$-Ar), 2.57–2.48 (m, 2H, CO-CH$_2$-CH$_2$-Ar), 2.27–2.17 (m, 4H, piperazine), 2.10–1.76 (m, 4H, O-CH$_2$-C*H*$_2$-C*H*$_2$-CH$_2$-Br).

1-(4-Benzylpiperazin-1-yl)-4-(4-(4-bromobutoxy)phenyl)butan-1-one (**16**)

Yellow oil; yield 60 %. IR (KBr, selected lines) cm^{-1}: 3430, 2927, 1645, 1510, 1433, 1242, 1177, 1030, 746. ^{1}H NMR (200 MHz, CDCl$_3$): δ 7.48–7.30 (m, 5H, aromatic), 7.08 (d, *J* = 8.6 Hz, 2H, aromatic), 6.80 (d, *J* = 8.6 Hz, 2H, aromatic), 3.97 (t, *J* = 5.8 Hz, 2H, O-C*H*$_2$-CH$_2$-CH$_2$-CH$_2$-Br), 3.73 (s, 2H + 2H, Ar-C*H*$_2$-N + piperazine), 3.57–3.46 (m, 2H + 2H, O-CH$_2$-CH$_2$-CH$_2$-C*H*$_2$-Br, piperazine), 2.60 (t, *J* = 7.3 Hz, 2H + 4H, CO-CH$_2$-CH$_2$-C*H*$_2$-Ar + piperazine), 2.28 (t, *J* = 7.4 Hz, 2H, CO-C*H*$_2$-CH$_2$-CH$_2$-Ar), 2.15–1.81 (m, 2H + 4H, CO-CH$_2$-C*H*$_2$-CH$_2$-Ar, O-CH$_2$-C*H*$_2$-C*H*$_2$-CH$_2$-Br).

Table S1. HO-1 inhibition and binding properties of hybrids **1–4** and reference compounds.

**Compd**	**n**	**IC$_{50}$ HO-1 (μM)**	**σ$_1$R K_i (nM) (%)[1]**	**σ$_2$R K_i (nM) (%)[1]**
**1**	0	229.91 ± 5.1	> 10,000 (90)	> 10,000 (54)
**2**	1	215.59 ± 3.6	> 10,000 (73)	> 10,000 (67)
**3**	2	161.92 ± 1.8	> 10,000 (78)	> 10,000 (66)
**4**	3	87.72 ± 1.3	> 10,000 (55)	4,624 ± 663
**LS0[2]**		2.1 ± 0.3[3]	--	--
**LS4/28[2]**		0.9 ± 0.08[3]	--	--
**LS6/42[2]**		0.95 ± 0.02[3]	--	--
**Azalanstat**		5.30 ± 0.4[3]	---	
**SI1/13[2]**		--	1.6 ± 0.05[4]	1,418 ± 18[4]
**RFB/13[2]**		--	8.8 ± 0.22[4]	3,253 ± 40[4]
(+)-Pentazocine		--	4.3 ± 0.5	1,465 ± 224
DTG		--	124 ± 19	18 ± 1
Haloperidol		--	2.6 ± 0.4	77 ± 18

[1] % of inhibition at 10 μM
[2] Figure 1 main text.
[3] Data taken from [1-3].
[4] Data taken from [4, 5].

Table S2. Elemental analysis data for compounds **1–4**.

				**Calcd**			**Found**	
**Compd**	Formula	Mw	C	H	N	C	H	N
**1**	$C_{25}H_{30}N_4O_2$	418.4	71.74	7.23	13.39	71.56	7.21	13.42
**2**	$C_{26}H_{32}N_4O_2$	432.7	72.19	7.46	12.95	71.98	7.44	12.99
**3**	$C_{27}H_{34}N_4O_2$	446.0	72.62	7.67	12.55	72.53	7.66	12.58
**4**	$C_{28}H_{36}N_4O_2$	460.2	73.01	7.88	12.16	72.91	7.87	12.20

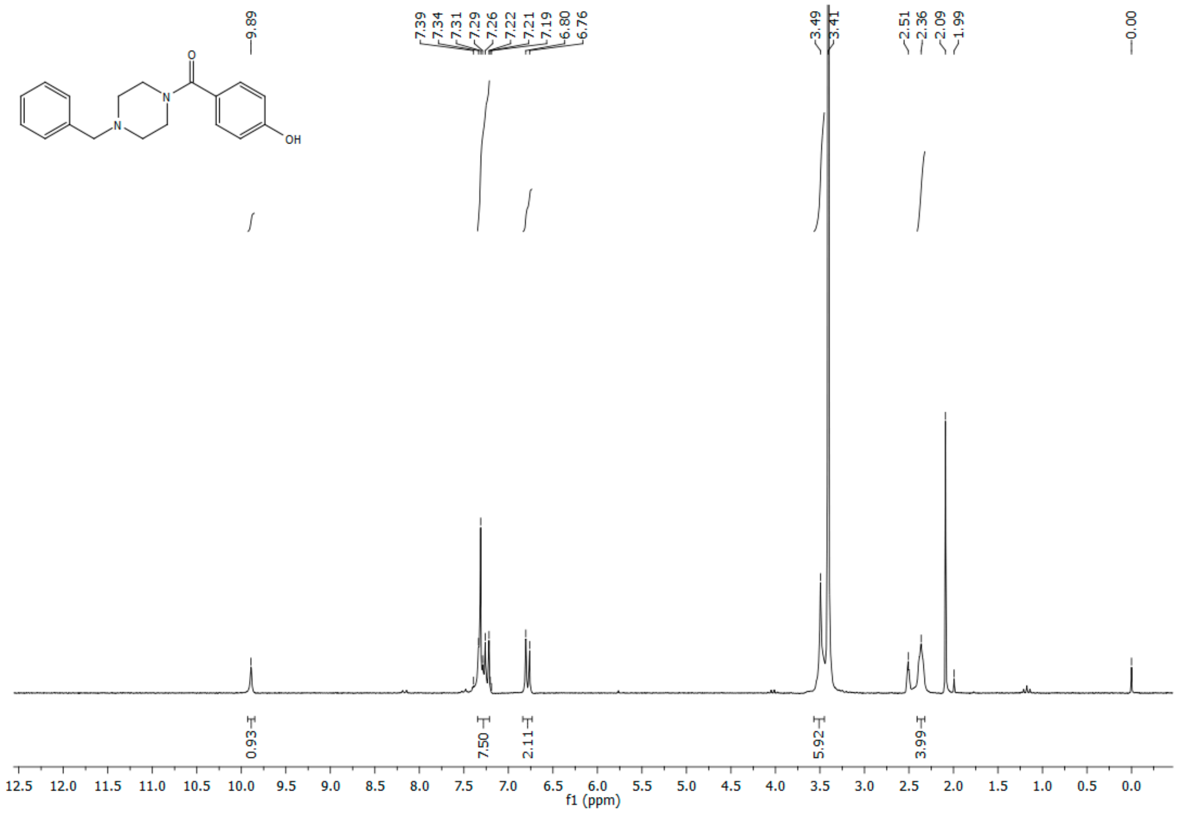

Figure S1. ^{1}H NMR of compound **9.**

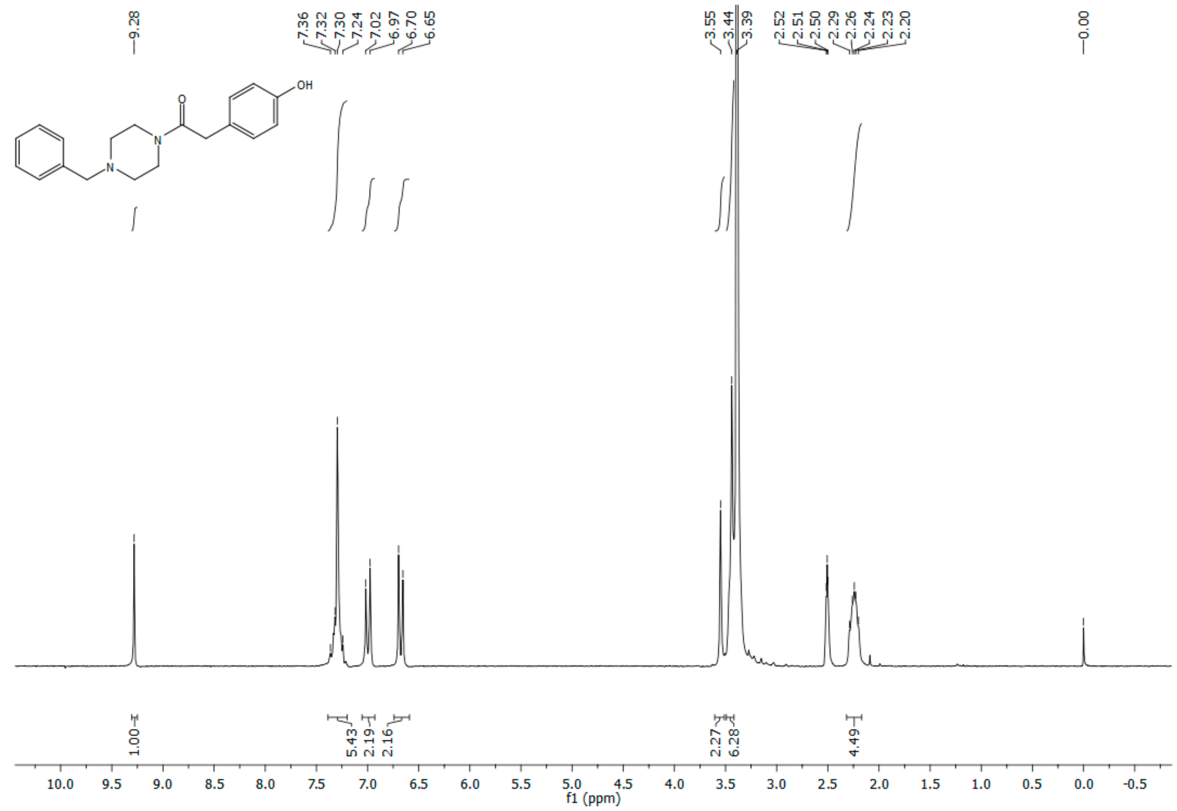

Figure S2. ^{1}H NMR of compound **10.**

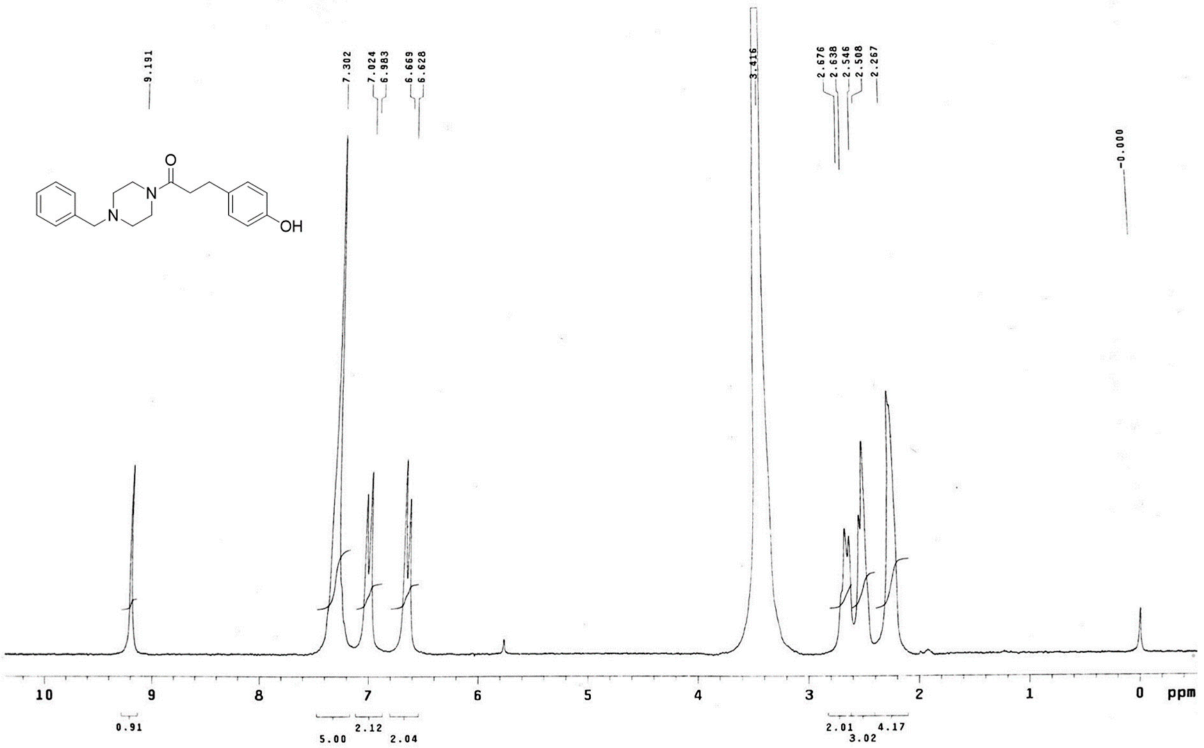

Figure S3. ^{1}H NMR of compound **11.**

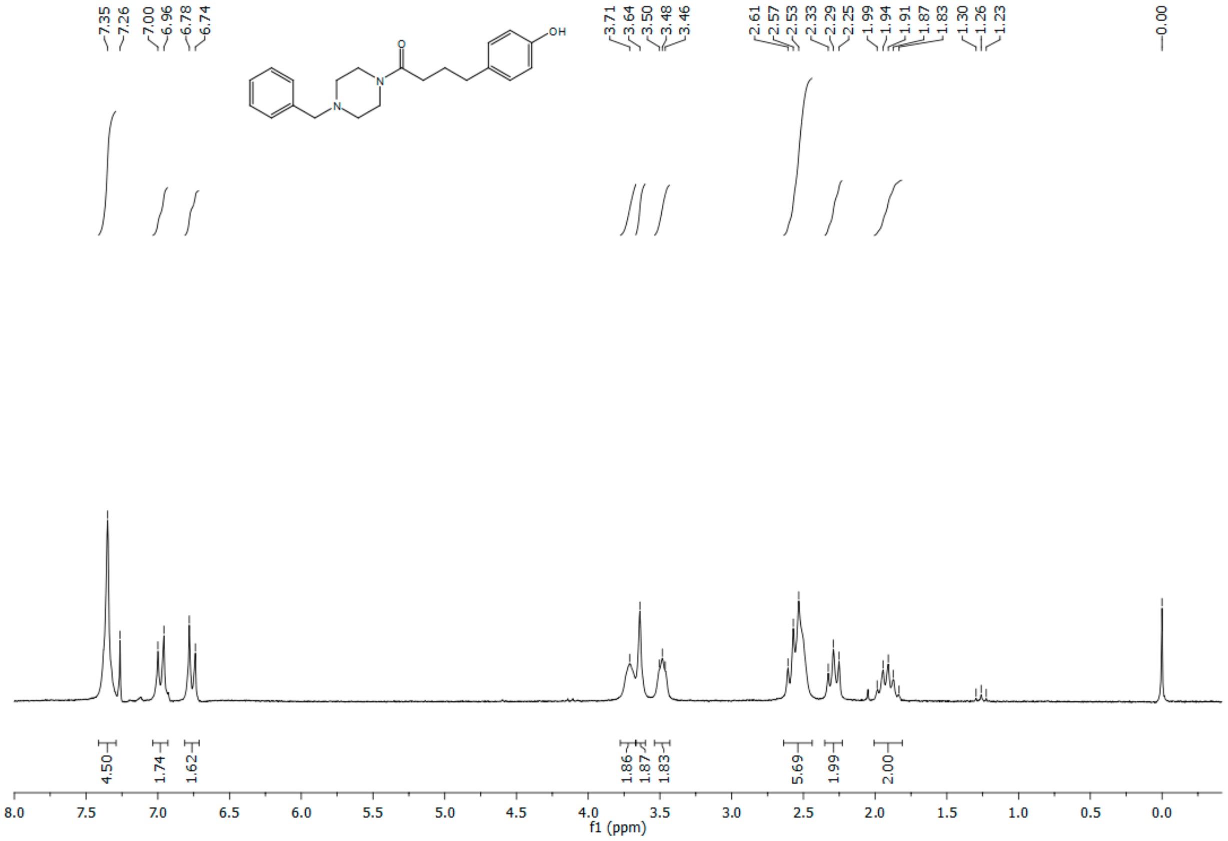

Figure S4. ^{1}H NMR of compound **12.**

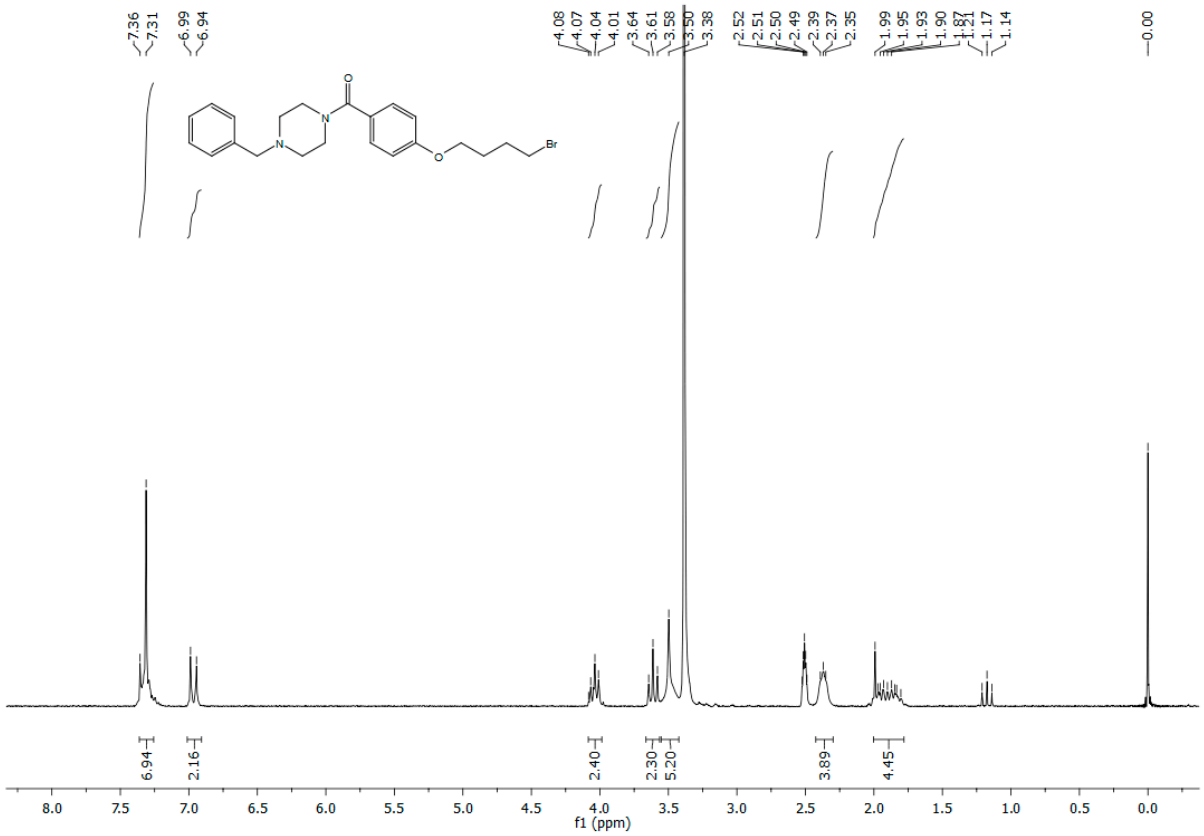

Figure S5. ^{1}H NMR of compound **13.**

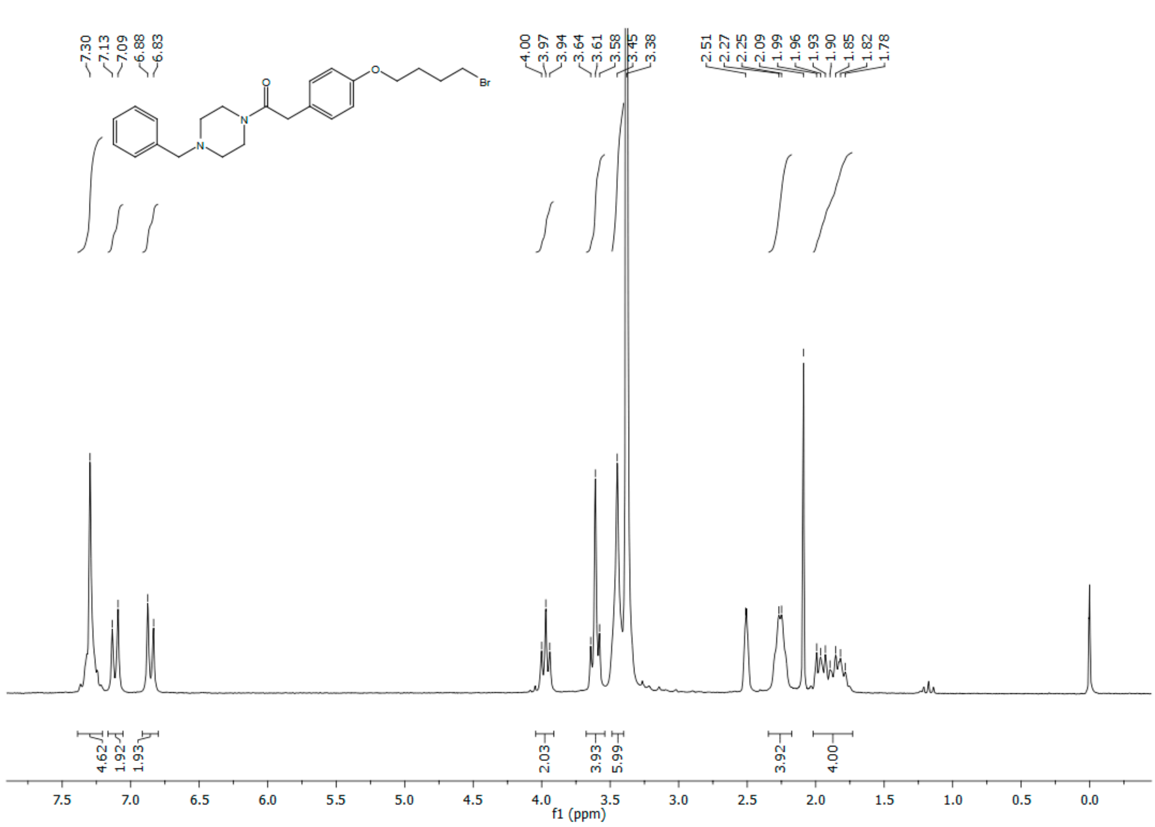

Figure S6. ^{1}H NMR of compound **14.**

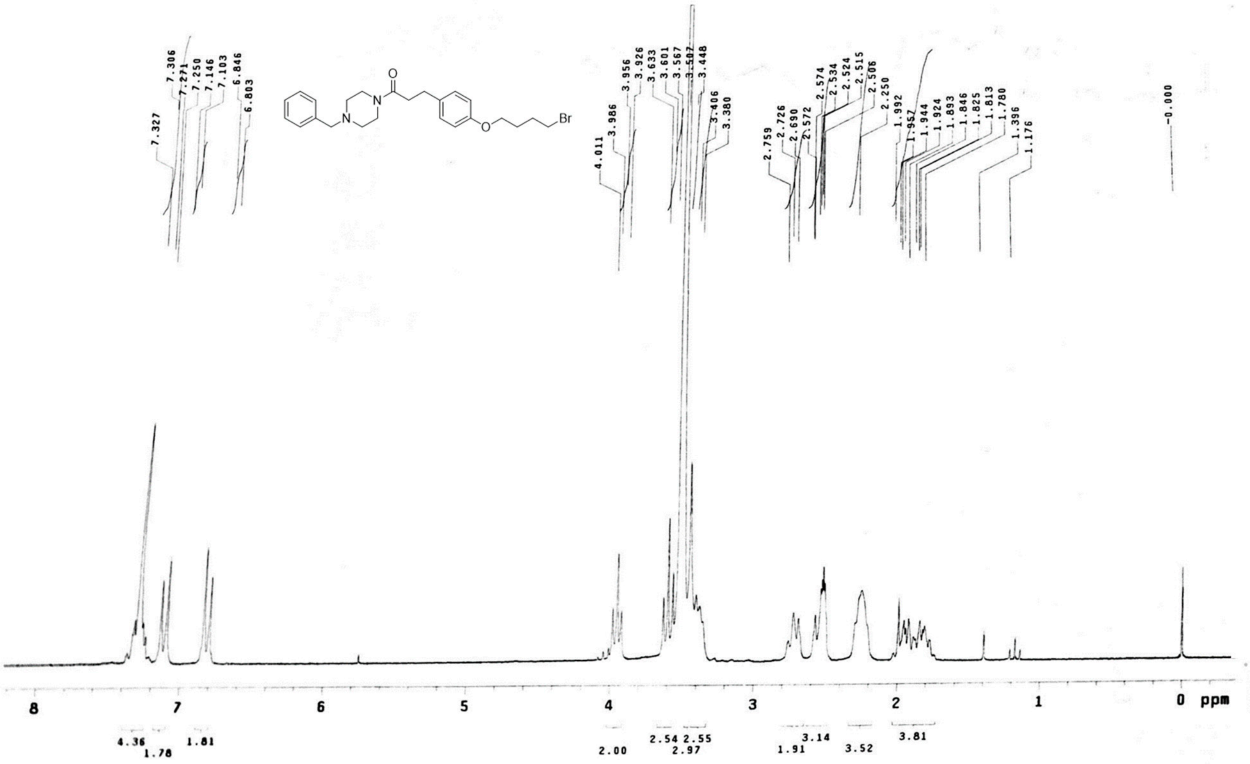

Figure S7. ^{1}H NMR of compound **15.**

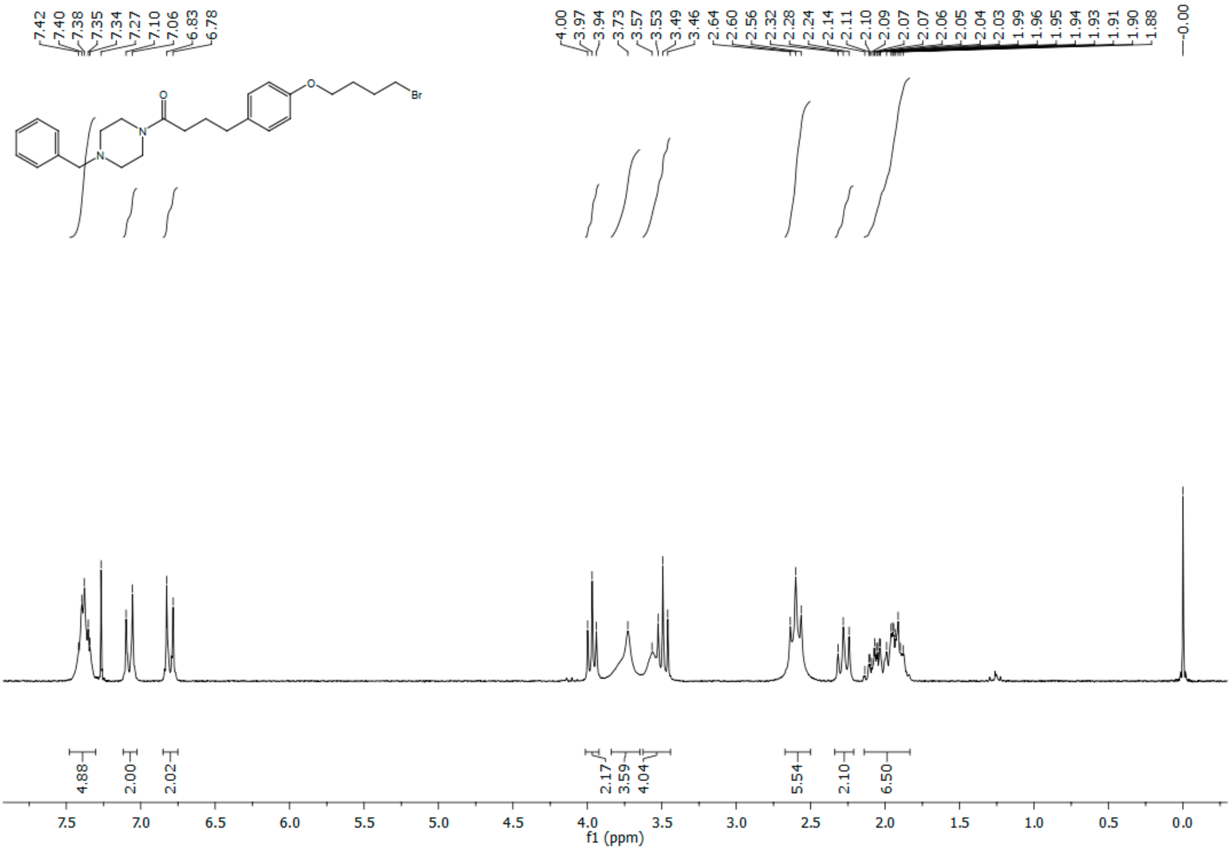

Figure S8. ^{1}H NMR of compound **16.**

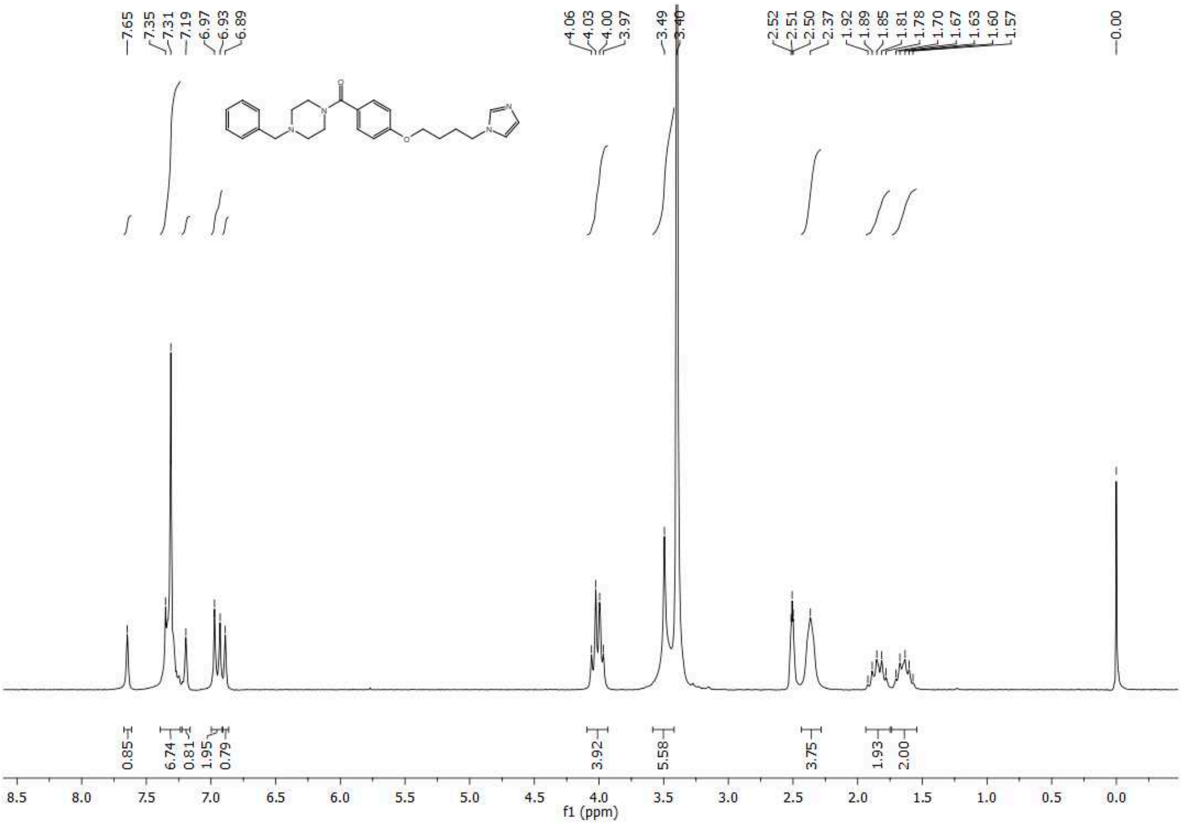

Figure S9. ^{1}H NMR of compound **1.**

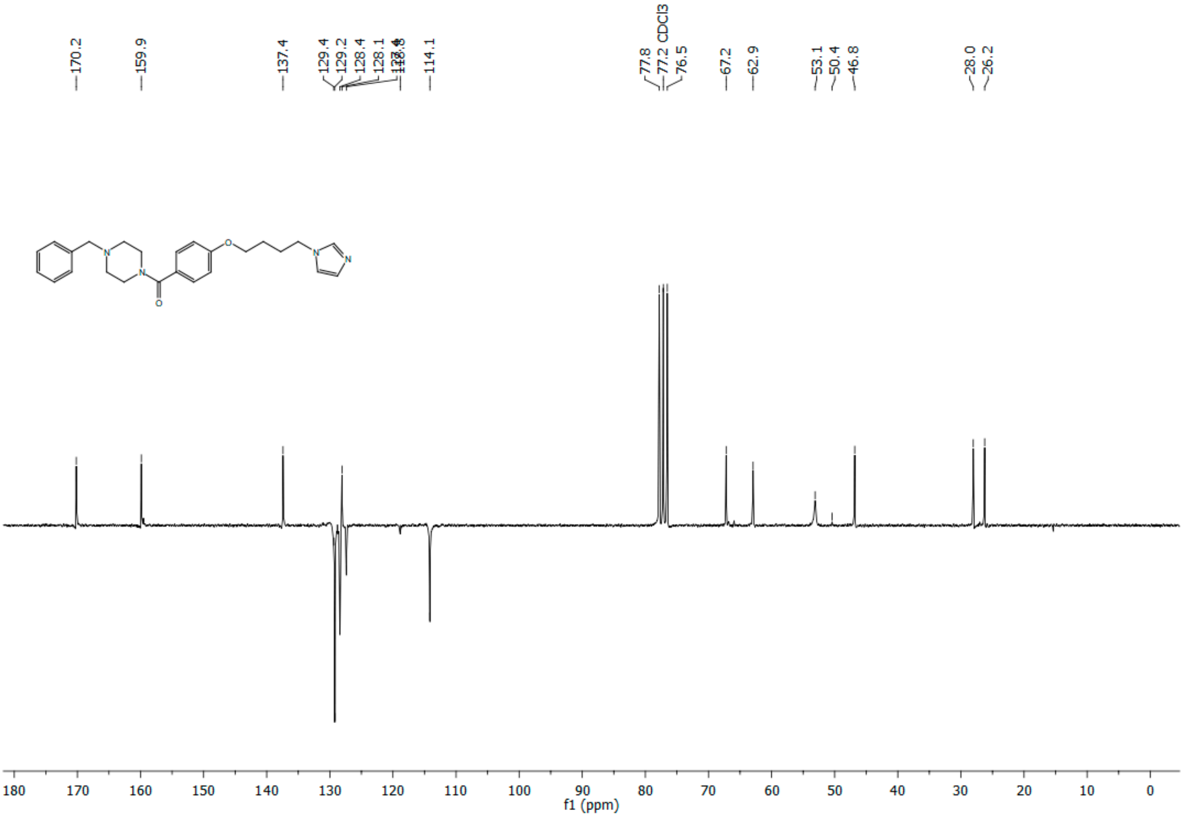

Figure S10. ^{13}C NMR of compound **1.**

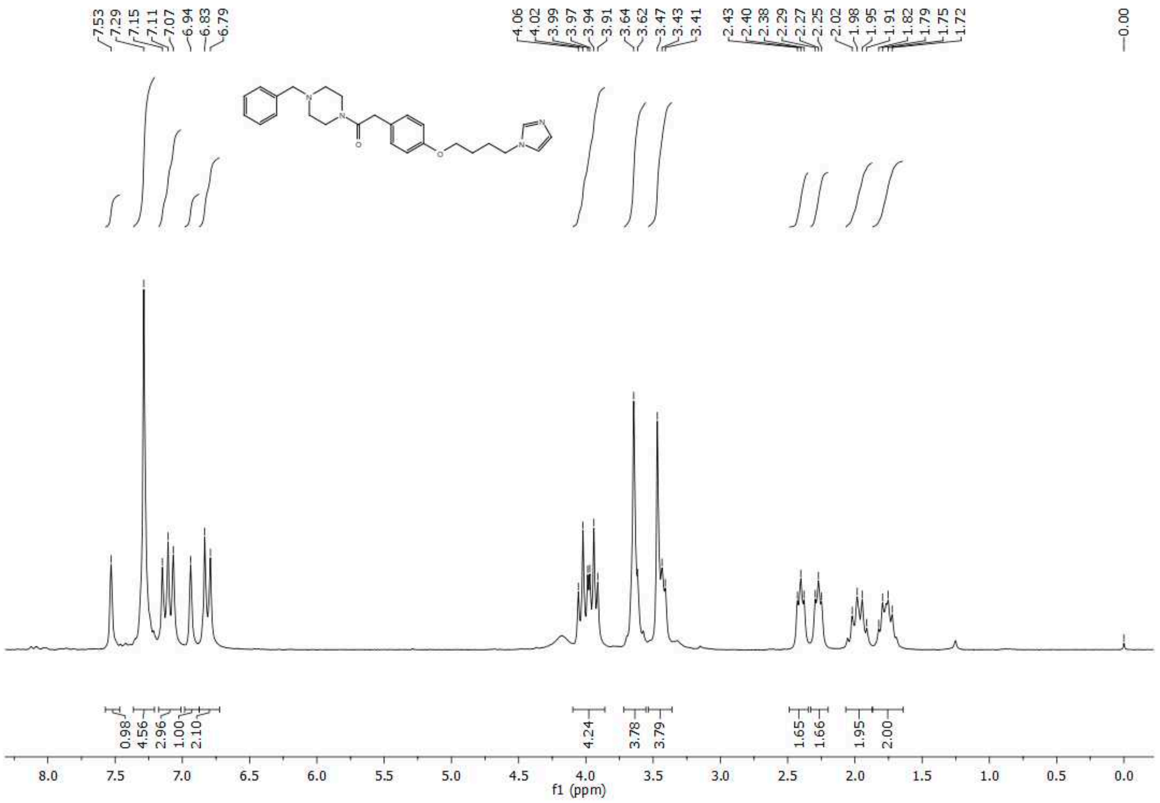

Figure S11. ^{1}H NMR of compound **2.**

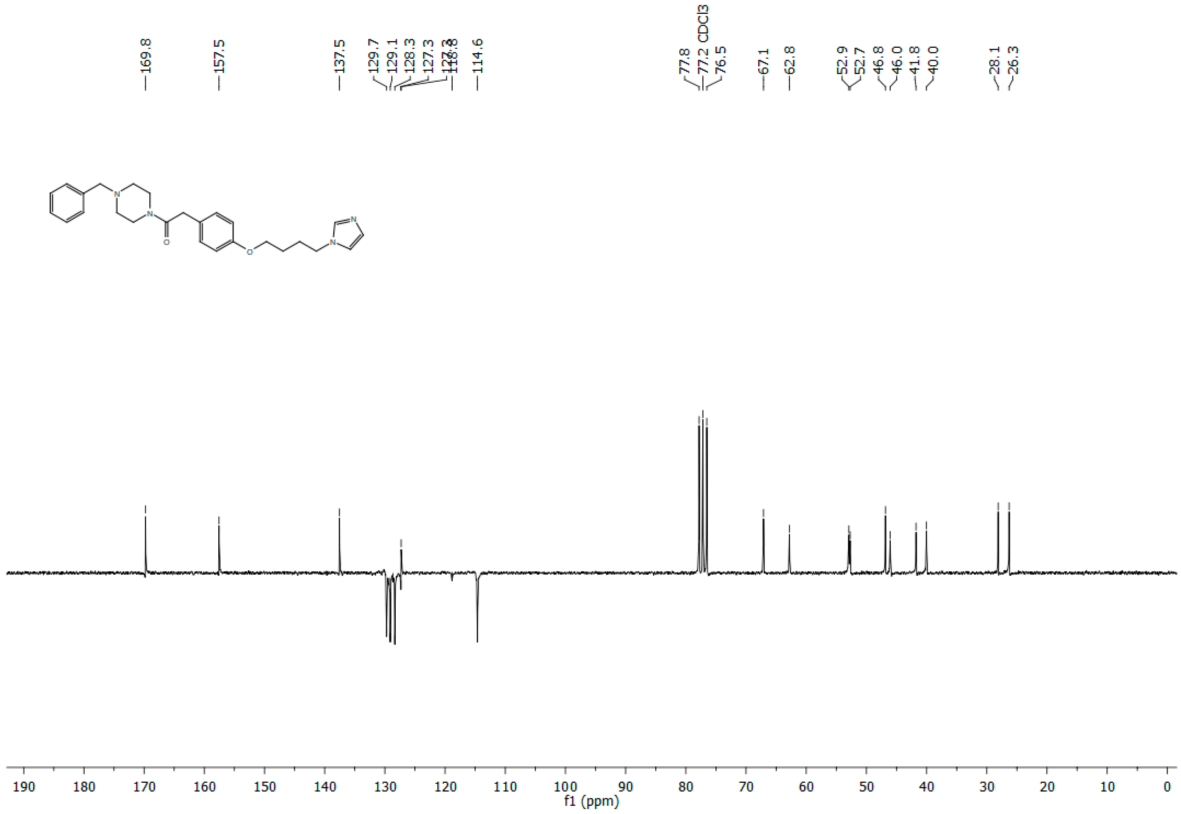

Figure S12. ^{13}C NMR of compound **2.**

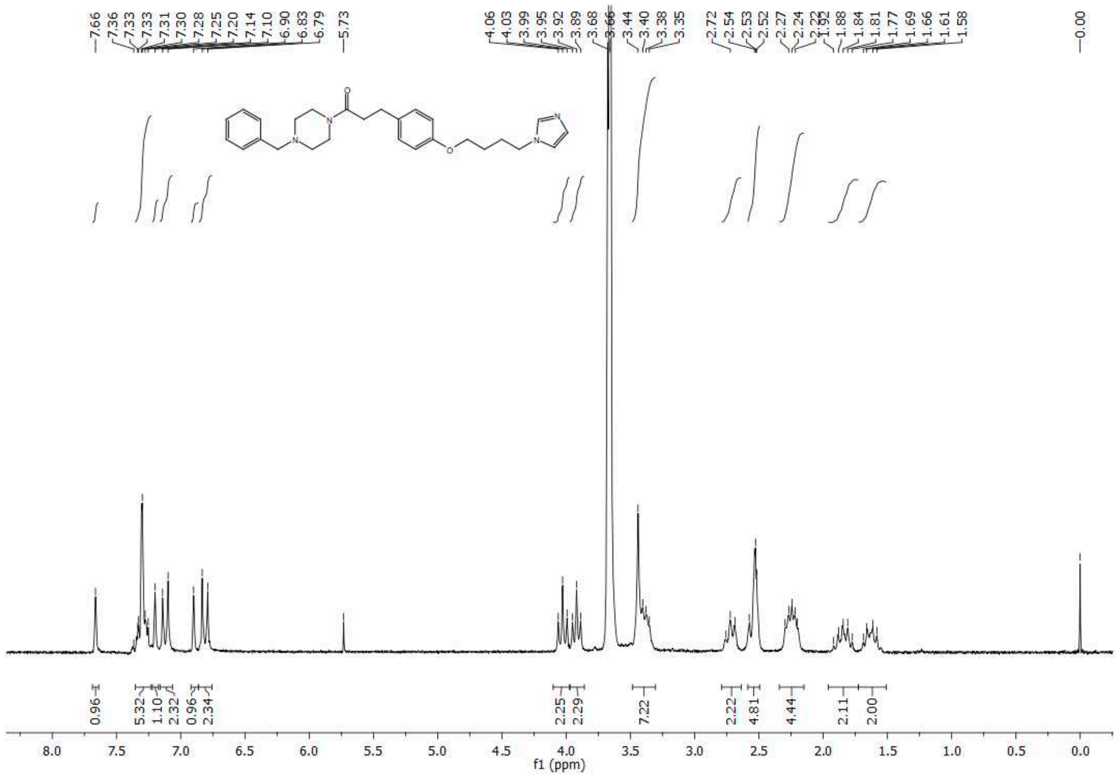

Figure S13. ^{1}H NMR of compound **3.**

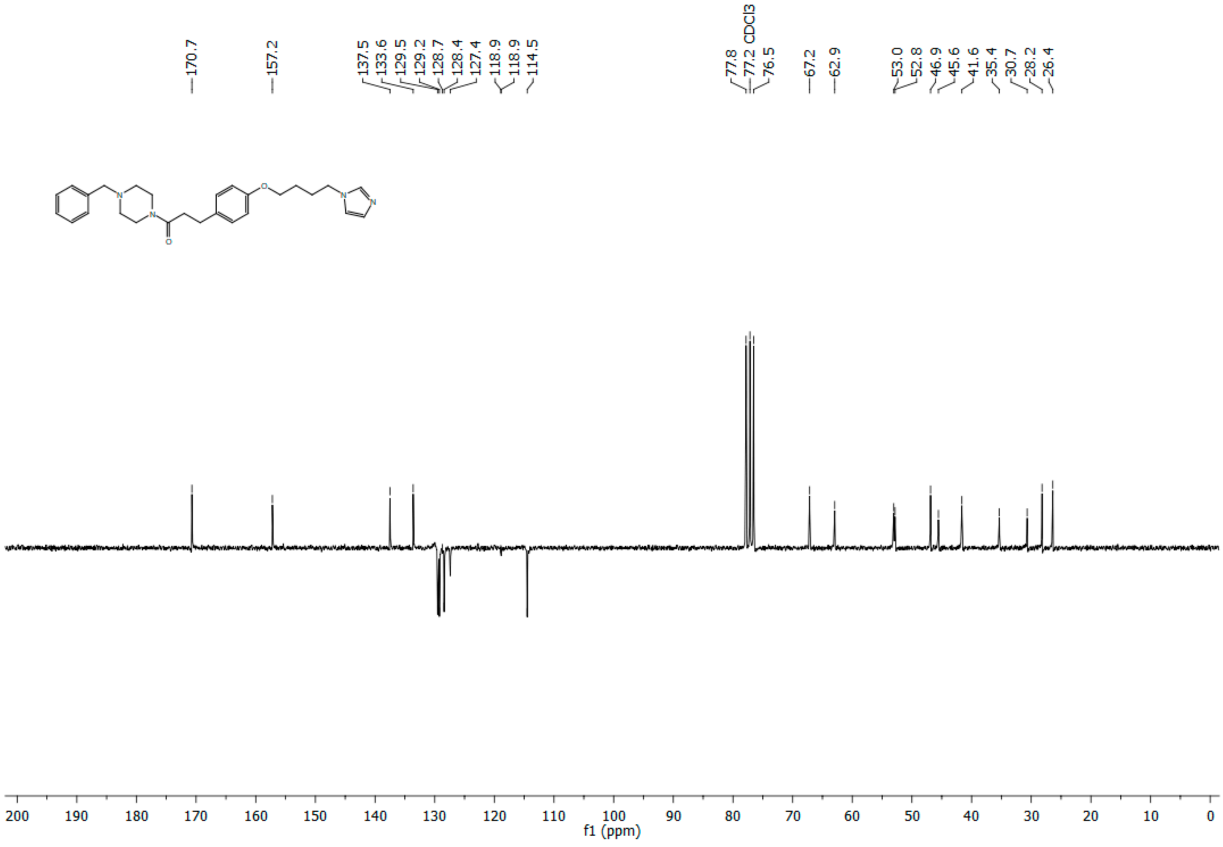

Figure S14. ^{13}C NMR of compound **3.**

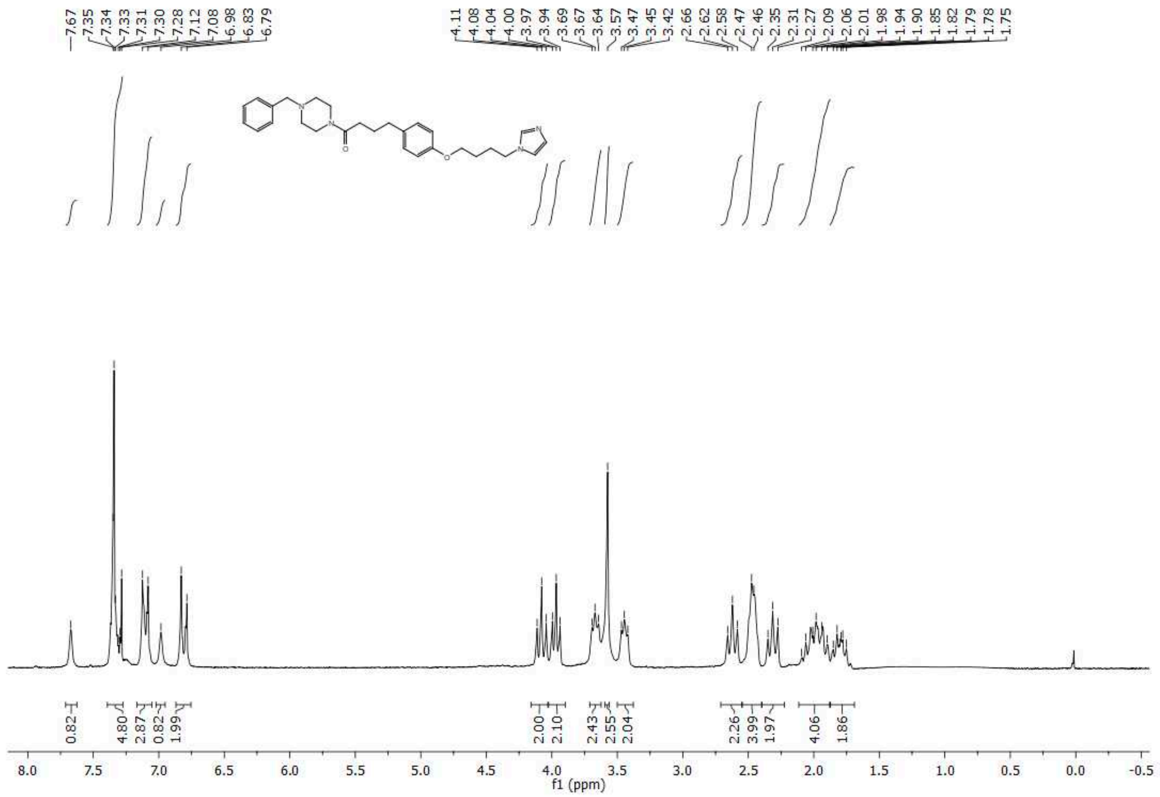

Figure S15. ^{1}H NMR of compound **4.**

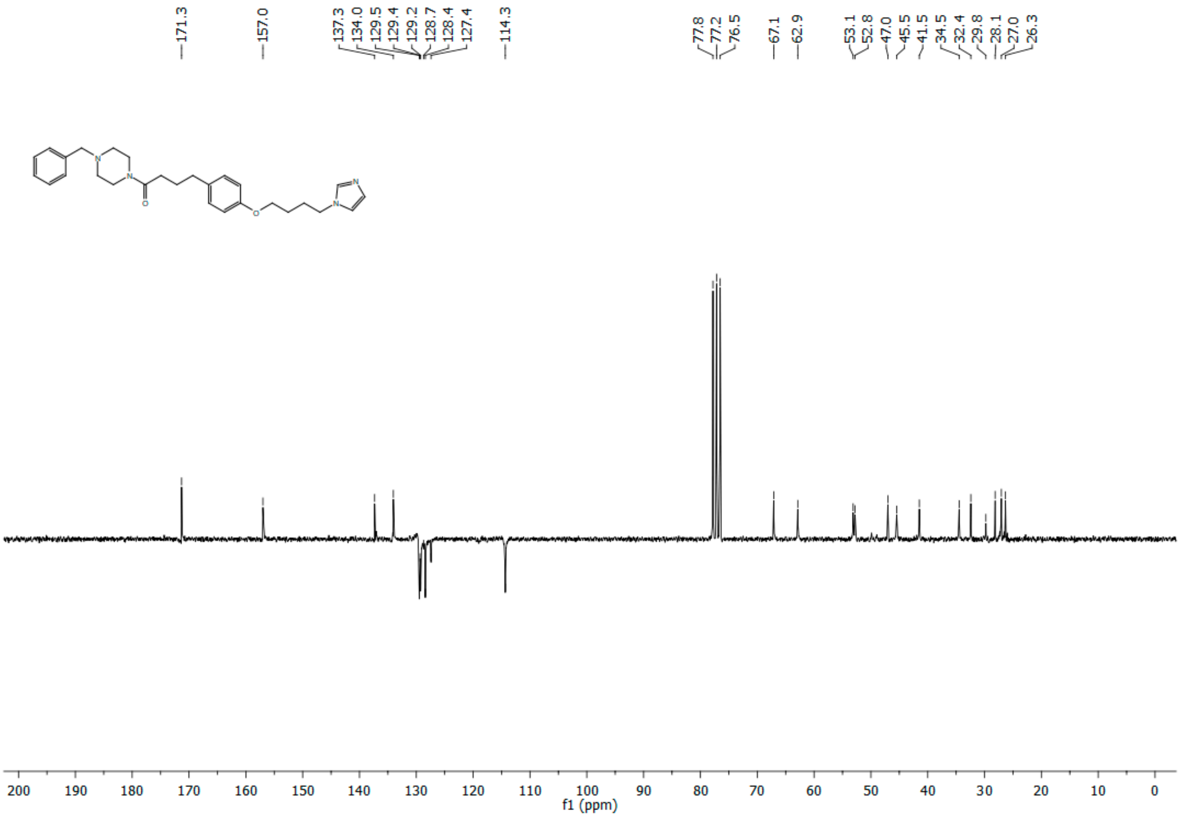

Figure S16. ^{13}C NMR of compound **4.**

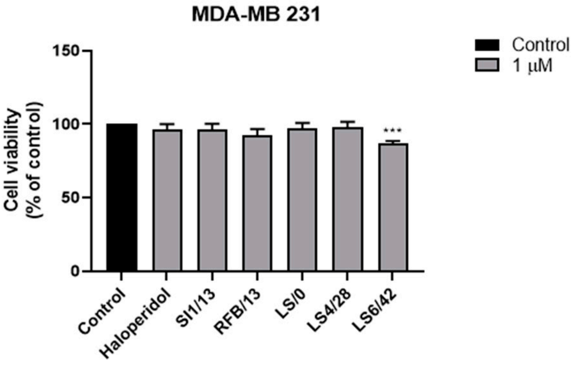

Figure S17. Effect of σR ligands haloperidol, **SI1/13** and **RFB/13** and of HO-1 inhibitors **LS/0**, **LS4/28** and **LS6/42** treatments on cell viability of MDA-MB 231 cell line, assessed by MTT assay at the doses of 1 μM. Results are representative of at least three independent experiments and values are expressed as percentage of control (% of control). Data represent means ± SEM. ***p<0.001 vs control as determined by One-way ANOVA followed by Tukey's multiple comparison test**.**

References

1. Salerno, L.; Pittala, V.; Romeo, G.; Modica, M. N.; Siracusa, M. A.; Di Giacomo, C.; Acquaviva, R.; Barbagallo, I.; Tibullo, D.; Sorrenti, V., Evaluation of novel aryloxyalkyl derivatives of imidazole and 1,2,4-triazole as heme oxygenase-1 (HO-1) inhibitors and their antitumor properties. Bioorg Med Chem. **2013,** *21*, 5145-5153.
2. Salerno, L.; Amata, E.; Romeo, G.; Marrazzo, A.; Prezzavento, O.; Floresta, G.; Sorrenti, V.; Barbagallo, I.; Rescifina, A.; Pittala, V., Potholing of the hydrophobic heme oxygenase-1 western region for the search of potent and selective imidazole-based inhibitors. Eur J Med Chem. **2018,** *148*, 54-62.
3. Greish, K. F.; Salerno, L.; Al Zahrani, R.; Amata, E.; Modica, M. N.; Romeo, G.; Marrazzo, A.; Prezzavento, O.; Sorrenti, V.; Rescifina, A.; Floresta, G.; Intagliata, S.; Pittala, V., Novel Structural Insight into Inhibitors of Heme Oxygenase-1 (HO-1) by New Imidazole-Based Compounds: Biochemical and In Vitro Anticancer Activity Evaluation. Molecules. **2018,** *23*.
4. Romeo, G. S., L.; Bonanno, F.; Pittalà, V.; Modica, M.N.; Siracusa, M.A.; Marrazzo, A.; Amata, E.; Arena, E.; Prezzavento, O., Synthesis of Novel Benzylpiperazine Derivatives as Ligands for the σ1 Receptor, Synthesis of Novel Benzylpiperazine Derivatives as Ligands for the σ1 Receptor. XXVI Congresso Nazionale della Società Chimica Italiana, Paestum (SA) **2017**.
5. Intagliata, S. S., L.; Pittalà, V.; Modica, M.N.; Arena, E.; Wilson, L.S; Mc Laughlin, J.P.; Prezzavento, O.; Romeo, G., Synthesis, binding, and functional properties of new benzylpiperazine derivatives as selective σ1 receptor ligands. Autumn Meeting for Young Chemists in Biomedical Sciences - Virtual conference. **2020**.
